# Extensive Geographic Mosaicism in Avian Influenza Viruses from Gulls in the Northern Hemisphere

**DOI:** 10.1371/journal.pone.0020664

**Published:** 2011-06-15

**Authors:** Michelle Wille, Gregory J. Robertson, Hugh Whitney, Mary Anne Bishop, Jonathan A. Runstadler, Andrew S. Lang

**Affiliations:** 1 Department of Biology, Memorial University of Newfoundland, St. John's, Newfoundland, Canada; 2 Wildlife Research Division, Environment Canada, Mount Pearl, Newfoundland, Canada; 3 Animal Health Division, Department of Natural Resources, St. John's, Newfoundland, Canada; 4 Prince William Sound Science Centre, Cordova, Alaska, United States of America; 5 Institute of Arctic Biology, University of Alaska Fairbanks, Fairbanks, Alaska, United States of America; Centers for Disease Control and Prevention, United States of America

## Abstract

Due to limited interaction of migratory birds between Eurasia and America, two independent avian influenza virus (AIV) gene pools have evolved. There is evidence of low frequency reassortment between these regions, which has major implications in global AIV dynamics. Indeed, all currently circulating lineages of the PB1 and PA segments in North America are of Eurasian origin. Large-scale analyses of intercontinental reassortment have shown that viruses isolated from *Charadriiformes* (gulls, terns, and shorebirds) are the major contributor of these outsider events. To clarify the role of gulls in AIV dynamics, specifically in movement of genes between geographic regions, we have sequenced six gull AIV isolated in Alaska and analyzed these along with 142 other available gull virus sequences. Basic investigations of host species and the locations and times of isolation reveal biases in the available sequence information. Despite these biases, our analyses reveal a high frequency of geographic reassortment in gull viruses isolated in America. This intercontinental gene mixing is not found in the viruses isolated from gulls in Eurasia. This study demonstrates that gulls are important as vectors for geographically reassorted viruses, particularly in America, and that more surveillance effort should be placed on this group of birds.

## Introduction

Influenza A viruses, in the family *Orthomyxoviridae*, are enveloped and possess a genome consisting of eight unlinked segments of negative-sense single stranded RNA [1], [2]. Wild birds are believed to be the primary reservoir for influenza A viruses, but they also have the capacity to infect a number of other host species [1], [3], [4]. Influenza A viruses have dynamic evolutionary capabilities with genetic changes occurring through mutation and through genome segment reassortment after coinfection with two or more viruses [1], [5], [6]. Most identified strains of avian influenza A viruses (AIV) are low pathogenic (LP), which are carried without readily apparent clinical signs. Highly pathogenic (HP) strains can cause significant morbidity and mortality in both wild birds and poultry [7]. LPAI viruses have been isolated from at least 105 wild bird species across 26 different families, although the highest prevalence of infection occurs in *Anseriformes* (ducks, geese, and swans) and *Charadriiformes* (shorebirds, gulls and terns) [8].

AIV are broadly divided into two clades, American and Eurasian [1], [8], believed to be a result of limited overlap in American and Eurasian bird ranges [8]. However, movement of viruses between these regions does occur because viruses with American sequences are found in Eurasia, and vice versa [9], [10], [11]. Unlike waterfowl, many gulls undergo intercontinental, pelagic and intracontinental movements [12]. It has been recognized that AIV sequences from gulls frequently form different clades than those isolated from other wild bird hosts [10], [13], [14], [15], including the hemagglutinin subtypes H13 and H16 that have been characterized as gull-specific [16], [17], [18]. Large-scale analyses of AIV genome sequences have demonstrated a low frequency of intercontinental reassortment events across all wild bird hosts, but it has been suggested that all H13 and H16 viruses have genomes with a mosaic of geographic origins [9], [10].

Intercontinental movements of birds and virus segments have large implications for AIV population structure. It has been demonstrated that Eurasian segments have invaded and displaced previously circulating American lineages; indeed, all currently circulating PB1, PA, and H6 lineages in North America are of Eurasian origin [19], [20]. To clarify the role that gulls play in global AIV dynamics we have sequenced the complete genomes of six AIV from Glaucous-winged Gulls (*Larus glaucescens*) in Alaska, greatly increasing the genomic information available for this host group from that region of North America. Alaska has been identified as an important area for mixing of numerous bird species from Eurasia and North America [21] and also for inter-regional mixing of AIV [22], [23], [24]. Through analysis of these new AIV sequences and their integration with all other available gull AIV sequences, we have evaluated a)historical gull surveillance globally, b) the phylogeography of gull AIV, c) the notion of gull specific lineages on both local and global scales, and d) the frequency of intercontinental reassortment of AIV gene pools in gulls. Our analyses reveal a remarkably high frequency of geographic reassortants in the gull AIV isolated in America, including all six viruses characterized in this study. Seven of the eight segments from five of these Alaskan viruses from 2009 appear Eurasian in origin, further validating the idea that Alaska is important in intercontinental AIV movement. Furthermore, intercontinental exchange is not limited to the H13 and H16 viruses isolated in North America, but was also found in gull viruses with other HA subtypes. In contrast, intercontinental reassortment was not observed in the AIV isolated from gulls in Eurasia. This study demonstrates that gulls are important for geographic reassortment of AIV, and are likely one of the major host groups involved in the movement of AIV genes from Eurasia to America.

## Results

### Host, spatial and temporal trends in historical gull AIV identification

In addition to the six viruses sequenced in this study, partial and/or complete sequence information was available from 142 other AIV isolated from gulls globally between 1975 and 2009 (as of September 2010). There are numerous other viruses detected in and/or isolated from gulls that have not yet been sequenced [8], [9], [25], [26], [27], [28], [29].

**Figure 1:**
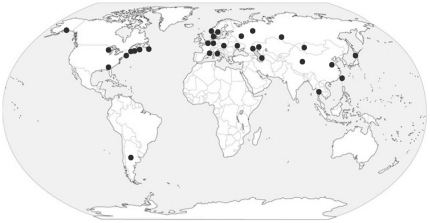
Locations of gull AIV identifications for which sequence data are available, 1975–2009.

The AIV analyzed in this study were isolated from numerous different gull species with different migratory patterns, feeding behaviours, and life history strategies, and which are therefore representative of the diverse spectrum within the family *Laridae*, even though uniform global geographic representation is not present (Table 1) [30]. There are biases towards certain species, such as Black-headed Gulls (*Chroicocephalus ridibundus*) and Great Black-headed Gulls (*Larus icthyaetus*) that have been targeted in the Volga Delta, Russian Federation [31], [32], and American Herring Gulls (*L. smithsonianus*) and Laughing Gulls (*L. atricilla*) that have been targeted on the Atlantic coast of the United States (Table 1). Therefore, the majority of sequenced viruses from North America are from the eastern coast (55 of 72), and only 14 viruses isolated in Alaska, including the six sequenced in this study, represent the Pacific coast. Viruses have been sequenced from more varied locations in Eurasia, but most coastal regions of Eurasia remain unrepresented (Figure 1). A single gull AIV from South America has been sequenced, and there are no gull viruses available from Australia or Africa (Figure 1).

**Table 1:** Species in the family *Laridae* from which AIV sequence data are available.

Species	Number of viruses
Black-headed Gull (*Chroicocephalus ridibundus*)^1^	16
Black-legged Kittiwake (*Rissa tridactyla*)	1
Brown-headed Gull (*Chroicocephalus brunnicephalus*)	6
Common Gull (*Larus canus*)	2
Glaucous Gull (*Larus hyperboreus*)	7
Glaucous-winged Gull (*Larus glaucescens*)	6
Great Black-backed Gull (*Larus marinus*)	1
Great Black-headed Gull (*Larus icthyeatus*)	18
American Herring Gull (*Larus smithsonianus*)	15
Herring Gull (*Larus argentatus*)	9
Kelp Gull (*Larus dominicanus*)	1
Laughing Gull (*Larus atricilla*)	22
Little Gull (*Larus minutus*)	1
Mediterranean Gull (*Larus melanocephalis*)	1
Mongolian Gull (*Larus vegae mongolicus*)	2
Ring-billed Gull (*Larus delawarenis*)	5
Sabine's Gull (*Larus sabini*)	1
Slaty-backed Gull (*Larus schistisagus*)	1
Slender-billed Gull (*Larus genei*)	1
Yellow-legged Gull (*Larus michahellis*)	1
Unknown gull species	31

1. Black-headed Gull has recently been reclassified from *Larus ridibundus* to *Chroicocephalus ridibundus* as proposed by Pons et al (2005).

The availability of AIV sequences also is not uniform along a temporal scale, and virus isolations are clustered within specific time periods (Table 2). The majority of gull viruses sequenced in the Americas were isolated between 1985–1989 and after 2000, and only 2 partial genome sequences are available from viruses identified during the 1990s. In Eurasia, there has been steady isolation and sequencing of viruses from the Volga River Delta, Russian Federation [33], [34]. The number of virus sequences has also increased since 2000, partially due to mass mortality events associated with H5N1 outbreaks in China [35], [36]. Work with gulls in Europe in 1999 and 2000 was particularly important and led to the description of a new subtype, H16, from European Black-headed Gulls [18].

**Table 2:** Availability of gull AIV sequence data by virus identification date.

Year	Region	
	America	Eurasia
1975–1979	7	7
1980–1984	5	8
1985–1989	23	6
1990–1994	1	2
1995–1999	1	7
2000–2004	12	15
2005–2009	17	37
Total (148)	66	82

### Subtype diversity and distributions

The distribution of AIV sequences between America and Eurasia is fairly even (Table 2). Of the 148 AIV with sequence data available, many only have sequence information available for the HA segment, particularly amongst the Eurasian viruses. Approximately half have sequence data available for the segments other than HA and NA: PB2 (n = 70), PB1 (n = 73), PA (n = 72), NP (n = 81), M (n = 84) and NS (n = 77) (Table S4), and 57 viruses have complete genome sequences available. Nearly half of the Eurasian sequences are HPAI H5N1 viruses (Table S4).

**Figure 2:**
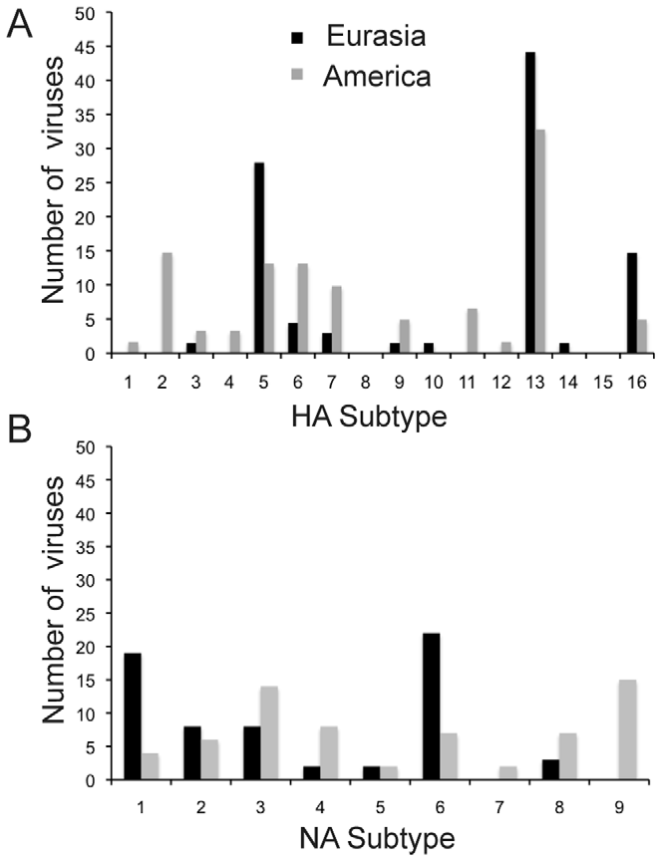
Subtype diversity within AIV from gulls in America and Eurasia. A. Distribution of hemagglutinin types. B. Distribution of neuraminidase subtypes.

All HA subtypes have been found in AIV from gulls, with the exceptions of H8 and H15. Viruses from Eurasia and America have different HA subtype trends, with the Eurasian viruses dominated by H5, H13 and H16, whereas the American gull viruses have higher subtype diversity and more H2, H6 and H7 viruses (Figure 2A).

All NA subtypes have been found in gull viruses in America, while N7 and N9 have not been detected in Eurasia (Figure 2B). Overall, the most common subtypes identified in gulls (excluding H5N1) are H13N6, H16N3, H13N2 and H13N9, whereas most other subtype combinations have only been identified three or fewer times (Table S5).

### Phylogeography of gull AIV

**Figure 3:**
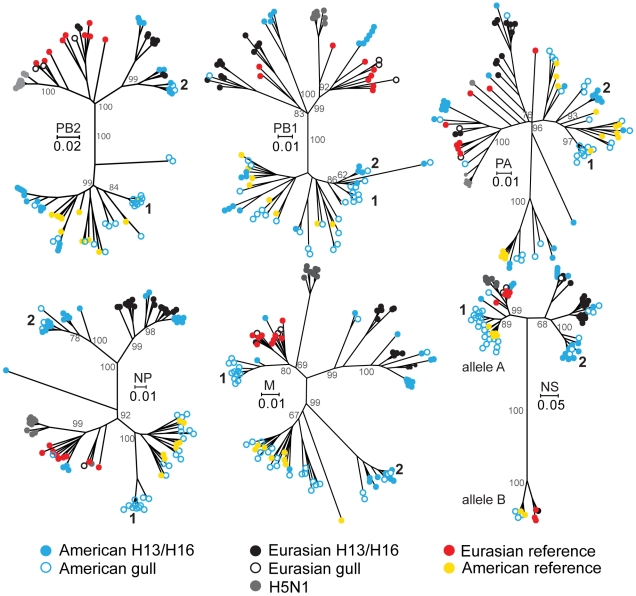
Neighbour-joining trees of PB2, PB1, PA, NP, M, and NS segments. Circles at the ends of branches identify the viruses as follows. Closed circles represent gull H13 and H16 viruses and open circles represent all subtypes except H13, H16 and H5N1. Blue represents gull viruses isolated in America and black those isolated in Eurasia. Closed grey circles represent H5N1 gull viruses. Yellow and red closed circles represent reference sequences from America and Eurasia, respectively (Table S3). The groups denoted with 1 and 2 on each panel consist of viruses that consistently group together (identified in Table S6). Scale bars indicate the number of substitutions per site. Trees with full virus identification labels and corresponding annotations are available in Figures S1, S2, S3, S4, S5, S6. All gull virus sequences used in this study are identified in Table S2.

Our phylogenetic analyses with individual segment sequences have provided new insights into AIV dynamics in gulls. As with other AIV, there is a phylogeographic pattern for gull AIV, with many viruses isolated in Eurasia and America on opposite sides of the trees (Figure 3). However, numerous American gull viruses are reassorted and thus group with the Eurasian viruses (Figure 3). It has previously been demonstrated that there are gull-specific clades [8], however some gull virus sequences are also integrated amongst viruses isolated from other host groups (Figure 3). Gull-specific lineages of the M segment are more similar to avian clades of the same geographic origin, with the exception of one American gull clade that is most similar to Eurasian avian sequences (Figure 3, group 1). On the contrary, the Eurasian and American gull-specific clades of the NP and NS trees are more similar to each other than they are to the avian clades from their respective regions (Figure 3). The American gull-specific clades are dominated by AIV from the 1980s, whereas the viruses isolated more recently are found in both gull-specific lineages and those containing waterfowl reference sequences (Figures S1, S2, S3, S4, S5, S6; Table S6). BLAST analyses of the sequences in these gull-specific clades verify the absence of a close relationship to any waterfowl viruses. However, BLAST analyses do indicate that some viruses from shorebirds, particularly Ruddy Turnstones (*Arenaria interpres*), are highly similar to the gull-specific groups 1 (up to 15 shorebird viruses) and 2 (up to 9 shorebird viruses). The polymerase-encoding segments (PB1, PB2 and PA) do not have clearly defined gull clades, but rather the gull viruses are generally integrated with other AIV sequences. All but four of the 77 NS gull sequences analyzed contain NS allele A (Figure 3; Figure S6).

Most of the North American gull AIV sequences are from Delaware Bay, and largely from the years 1986 to 1989. Our analyses show that there were two different virus lineages circulating in the gulls there at that time. One lineage comprised mostly H2 viruses (Figure 3, group 1; Figures S1, S2, S3, S4, S5, S6; Table S6) and the other H13 viruses (Figure 3, group 2; Figures S1, S2, S3, S4, S5, S6; Table S6). These two groups of viruses are distinct in five of the six trees (PB2, PA, NP, M, and NS). Although there are lower numbers of AIV sequences from subsequent years, it appears that some descendant segments of each lineage still persist because virus segments found more recently group with these sequences from the 1980s.

**Figure 4:**
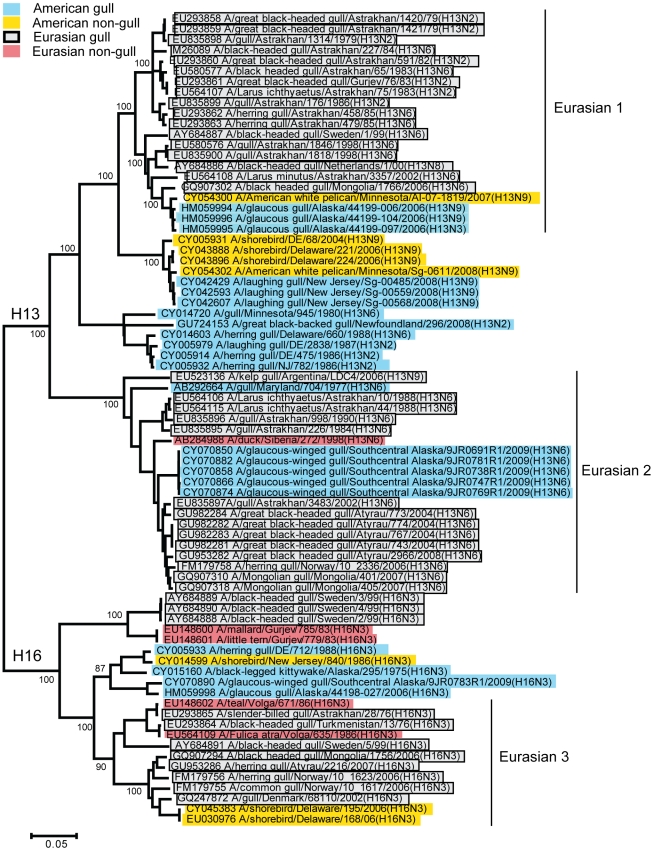
Neighbour-joining tree of all complete H13 and H16 sequences. Gull viruses isolated in Eurasia are highlighted in gray and those isolated in America in blue. Yellow and red highlight viruses from other bird species from America and Eurasia, respectively. The scale bar indicates the number of substitutions per site. Bootstrap values are provided as percentages based on 10000 replicates for selected major branch points.

It has been proposed that the genetic structure of AIV is in part a result of transient genetic linkage between PB2, PB1, PA, NP, and M segments and the HA and NA, and perhaps NS, segments [37]. Our data support such a linkage because the H13 segments (Figure 4, American clade 1980–1989) of the group 2 viruses indicated on Figure 3 (also Figures S1, S2, S3, S4, S5, S6; Table S6) are similar, and the H2 segments (Figure S7) of the group 1 viruses indicated on Figure 3 (also Figures S1, S2, S3, S4, S5, S6; Table S6) are similar. The M segments of the group 1 viruses indicated on Figure 3 (Figure S7; Table S6) are of Eurasian origin, and the H2 viruses within this group (Table S6) are more similar to Eurasian viruses than they are to American avian or other American gull H2 viruses (Figure S7). The PB2 segments of the group 2 viruses indicated on Figure 3 (Figure S1; Table S6) are also more similar to Eurasian viruses than they are to American avian or gull viruses.

Similar to the two different American clades of gull AIV found in Delaware Bay in the 1980s, there are two very different H13 HA lineages circulating in the gull population of the Caspian Sea, Russian Federation [31], [33], [34]. The Eurasian clade 1 is more similar to two clades of American viruses: one lineage that was circulating during the 1980s and one that contains viruses currently circulating (Figure 4). The Eurasian clade 2 is similar to two American viruses from the 1970s, as well as the five H13 viruses that were isolated in Alaska in 2009 and sequenced in this study. In contrast, three H13 viruses from 2006 in Alaska are within Eurasian clade 1.

Eurasian viruses dominate the H16 portion of the tree (Figure 4), with the exception of a single North American lineage that includes viruses from 1975, 1986, 1988, and the 2009 Alaskan H16 virus isolated in this study. Two additional H16 sequences from shorebirds in Delaware Bay in 2006 are within the Eurasian clade 3.

Analyses of the most common gull virus NA subtypes, N3 and N6, reveal that these sequences are largely separated into distinct North American and Eurasian lineages, but the presence of Eurasian sequences in the American viruses is evident for both (Figure S8). Indeed, all NA segments from the Alaskan viruses sequenced in this study, in addition to a sequence from Alaska in 2006, are more similar to sequences from Eurasian viruses than those from other gull viruses in America. These N3 sequences from the Alaskan gulls are also distinct from the most closely related Eurasian lineage (Figure S8A), suggesting poor sequence coverage of viruses from gulls and/or the existence of unique lineages of this gene in western North America. Analysis of the N6 sequences indicates there are two different Eurasian groups (Figure S8B).

### Pervasive inter-regional reassortment in gull AIV

**Figure 5:**
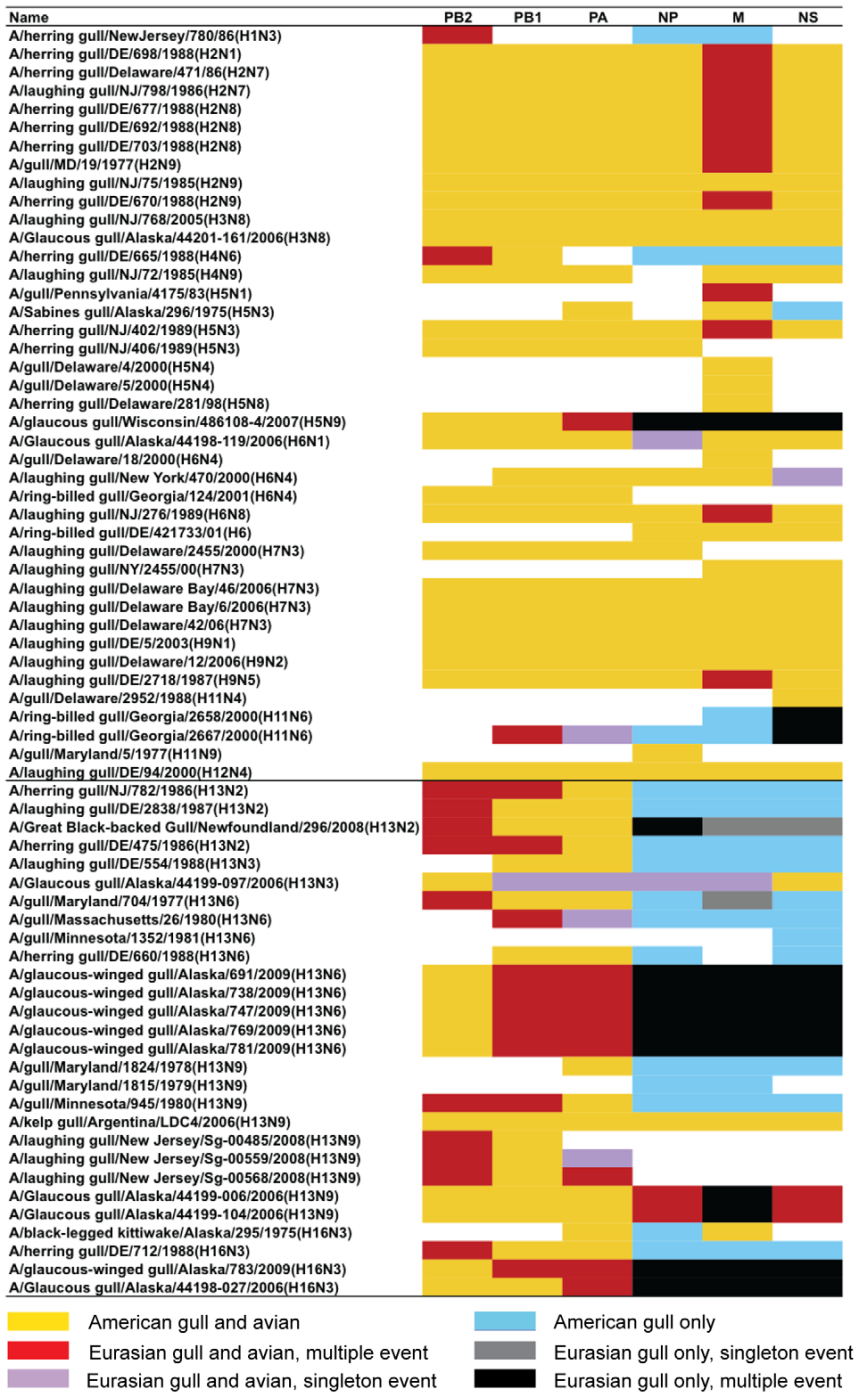
Geographical reassortment in AIV isolated from gulls in America. Each segment is represented by a box, and ordered by segment size from left to right (PB2, PB1, PA, NP, M, and NS). Orange boxes indicate clustering into a clade containing both avian and gull viruses (i.e. not geographically reassorted), red indicates the segment is within a clade of American viruses that all fall within a clade of Eurasian gull and avian viruses (i.e. multiple American occurrences of the Eurasian segment), and pink indicates the segment is from an American virus and falls within a clade containing only Eurasian gull and avian viruses (i.e. the Eurasian segment has been found in America only once). For the NP, M and NS segments where gull-only clades are well-supported, additional colors have been added as follows. Blue indicates clustering into a clade of American gull viruses, black indicates the segment is part of a group of American gull viruses that fall within a clade of Eurasian gull viruses, and grey indicates the segment falls within a clade containing only Eurasian gull viruses (i.e. the Eurasian segment has been found in America only once). Segments with partial sequence information available are included, but white boxes indicate no sequence information is available. The viruses are ordered by HA subtype, with a line between the H13/H16 subtypes and all other subtypes.

It was observed previously that the occurrence of geographical reassortment (i.e. between Eurasia and America) is much higher in the gull-specific H13 and H16 lineages compared to other AIV subtypes [10]. Our study indicates that, not only is reassortment prevalent in American H13 and H16 viruses, but also in other subtypes isolated from gulls in America (Figure 5). In contrast, there is no evidence of such reassortment in Eurasian gull viruses (Figures 3 and 4). A single Eurasian gull virus with an American segment has been identified, where the HA segment of an H9N2 virus isolated in southern France is of American origin [38].

Most of the gull viruses found in America contain segments with a mosaic phylogeographic pattern (Figure 5), with 40 of the 69 American viruses analyzed having at least one segment that falls within Eurasian lineages in phylogenetic analyses. Of the 28 viruses that did not contain geographically reassorted segments, 19 of the genomes were incomplete. Most notably, only one H13 virus from the Americas for which a complete genome sequence is available, A/kelp gull/Argentina/LDC4/2006(H13N9), is not geographically reassorted.

The Alaskan viruses from 2009 sequenced in this study contain the highest numbers of Eurasian segments ever found in viruses in North America. The five H13 viruses sequenced in this study each had seven Eurasian segments and the H16 virus had six. Five the 6 viruses sequenced from St. Lawrence Island, Alaska from Glaucous Gulls (*Larus hyperboreus*) in 2006 are also geographic reassortants [24].

## Discussion

Understanding of the pattern of AIV dynamics in gulls is still somewhat limited due to the small number of sequences available and poor uniformity of sequence availability over spatial, temporal and host species ranges. A more complete dataset would enable a better description of the introduction and extinction of lineages in this host group, and of reassortment events that are of great importance in AIV dynamics. Particularly lacking from the current data are viruses from the Southern Hemisphere. South Africa, for example, is significant in the history of AIV surveillance in wild birds, with the first documented case of HPAI in wild birds occurring there in Terns, a sister group of gulls, in 1959 (GU052814-GU052821) [39].

Despite large biases in the available dataset, studies of gull AIV reveal multiple factors affecting gull AIV dynamics, including differences in HA receptors, formation of gull specific clades, and the presence of partial genome constellations. There is a great diversity of HA subtypes in gulls, particularly in North America. The H13 and H16 hemagglutinin proteins are adapted for recognition of fucosylated sialyloligosaccharide receptors, which is required for virus attachment and hence efficient replication in gull intestinal cells [17], [40], and these subtypes are frequently found in gulls (Figure 2A). It has also been demonstrated that American gull H4 proteins are adapted for recognition to these receptors, increasing the fitness of these viruses in gulls [40].

The existence of gull-specific clades has previously been demonstrated [8], [15], [41], but we present the first comprehensive gull virus dataset analysis. This illustrates the presences of these clades in each of the PB2, PB1, PA, NP, M and NS (B allele) genes. Gull viruses, however, are not restricted to gull-specific clades as some gull virus sequences are integrated with those from other wild bird hosts. Furthermore, gull clades show different patterns between the polymerase-encoding segments and the NP, M and NS segments (Figure 3). Gull virus groupings for the PB2, PB1 and PA segments follow a geographical pattern where they are more similar to sequences from other wild bird hosts from the same geographical region and integrated into clades containing sequences from other wild bird hosts. In contrast, the NP and NS B allele segments of gull viruses form gull specific clades that are more similar between geographic regions than they are to those from wild bird hosts from the same geographical region. Further, gull-specific clades are not restricted to H13 and H16 gull subtypes (for example group 1 viruses on Figure 3; Table S6). The presence of gull-specific clades suggests that these forms have adaptations for replication in and/or transmission between gulls.

The presence of genome constellations, as previously proposed [10], are evident in Delaware Bay (groups 1 and 2 on Figure 3; Table S6), which may be the result of transient genetic linkage between the PB2, PB1, PA, and NP and the HA, NA and NS segments. If a favorable mutation occurs in the HA, NA or NS that results in an increase in the frequency of infection, the frequency of the internal segments of that particular strain will also increase in turn due to transient linkage, or “hitchhiking” [39]. Such transient genome constellations eventually dissociate due to high levels of reassortment and/or selective sweeps within the host populations [39]. These same two constellations each existed in Delaware Bay for at least 3 years, which we believe supports the linkage/hitchhiking phenomenon and indicates that the lengths of linkages can be multiple years in some circumstances. Furthermore, we have observed apparent linkages between H13 and N6, and H16 and N3, perhaps indicating that there is increased fitness in gulls for these subtype combinations.

The origin of Eurasian segments in North America has long been questioned, and previous studies have investigated ducks and shorebirds as potential vectors of these viruses [9], [42]. Gulls have been recognized to carry AIV with unique subtypes and geographic reassortants [16], [18], this is the first targeted analysis of all available gull virus sequences. Although Eurasian gulls do not seem to have AIV with American segments, analyses of the spatial, temporal and host species origin of the available sequences indicate poor coverage overall, especially in potentially important coastal regions. Based on the movement patterns of gulls, it might be predicted that if geographical reassortments of AIV were occurring in Alaska or eastern North America, the Eurasian segments would most likely originate in coastal regions including Kamchatka, Japan, China, Greenland, Iceland, Spain, Portugal and the United Kingdom [22]; unfortunately, there are currently no LPAI sequences available form gulls from these regions in Eurasia. There are few cases of geographic reassortant virus discovery in Eurasia overall, with the exceptions including viruses from a pelagic seabird, Common Murre [43], and individual ducks in Italy [44], India [45], and Japan [46]. Our analyses suggest that gulls are important mixing vessels for viruses and are possibly the main contributors of geographically reassorted viruses in North America. Most of the identified geographically reassorted segments appear to have resulted in successful invasion of North America because few of these sequences are similar to only Eurasian viruses; rather, most of these Eurasian sequences have been detected in North America more than once (Figure 5). Indeed, it is evident that some of these Eurasian lineages have diversified and are well established in North America, as is demonstrated for the group 1 M segment (Figure 3). Lastly, Alaska is indeed an important location to survey for Eurasian viruses [22], [23], [42], but more emphasis needs to be placed upon gulls in this location, and others. No AIV has been detected in North America with all eight segments of Eurasian origin, but our isolates suggest it might be possible that such a virus will be found in Alaskan gulls.

## Methods

### Ethics statement

This study was performed using a protocol approved by the Institutional Animal Care and Use Committee of the University of Alaska Fairbanks (Approval Number: 08-62).

### Virus isolation and characterization

Fecal samples were collected from Glaucous-winged Gull roosting sites at the city dock (60.547N, -145.785W), Hartney Bay (60.499N, -145.867W) and Odiak Slough (60.539N, -145.786W) in Cordova, Alaska between June and September 2009. Individual, freshly deposited feces from roosting locations used exclusively by this gull species were swabbed using a sterile-tipped applicator, which was then inserted into a tube containing M4RT viral transport media (VTM) (Remel, Lenexa, KS).

Screening for influenza A was performed using a two-step real-time RT-PCR approach [47]. Briefly, RNA was extracted from each swab sample using the MagMAX-96 Viral RNA Isolation Kit (Ambion, Austin, Texas) following the manufacturer's instructions. cDNA was synthesized using the M-MLV reverse transcriptase enzyme (Invitrogen, Carlsbad, California) and random hexamers (Invitrogen) and assayed for the presence of the AIV matrix gene by real-time PCR [48] using a TaqMan (Qiagen, Valencia, California) assay with a threshold cut-off (Ct) <40. Virus isolation was carried out in 9 to 11 day old SPF embryonated chicken eggs (Charles River, North Franklin, Connecticut) inoculated via the allantoic route. The eggs were candled daily to monitor for embryo mortality. Up to three blind passages were performed, and allantoic fluid was assayed for the presence of the avian influenza matrix gene after each passage as previously described [47].

For virus genome sequencing, cDNA was synthesized using the Uni12M primer [49] and the Superscript III First Strand Synthesis System for Reverse Transcriptase PCR (Invitrogen) with RNA extracted from the allantoic fluid. Twenty-eight PCR reactions were then carried out in order to amplify the entire genome using a combination of primers (Table S1) [15], [22], [46], [49], [50], [51], [52], [53], [54]. PCR products were purified using the QIAquick PCR Purification Kit (Qiagen). Capillary sequencing of PCR products was carried out at The Centre for Applied Genomics (Toronto, Canada). Complete segment sequences were assembled using Geneious v3.8.5 (Biomatters, New Zealand). Sequences generated in this study have been deposited in the NCBI GenBank database and the Influenza Resource Database under the accession numbers CY070847 – CY070894.

### Sequence analyses

For the analyses presented here, we included the full genome sequences we completed for six gull isolates from Alaska and sequences available at the National Centre for Biotechnology Information (NCBI) GenBank and Influenza Resource Databases [55]. All sequence data available from AIV isolated from gulls (as of April 2010) were retrieved (Table S2). Inconsistencies in virus names were resolved by checking associated publications when available. Viruses with more than one sequence per segment were further investigated, and sequences were compared using BLAST [56]; the most complete and/or most recent sequences were selected. Suspected language translation errors in host species names in GenBank were investigated to determine the correct host species based upon range distributions [57] and by translation of original foreign literature. Partial and complete sequences were considered when analyzing geographic assignment of segments and basic subtype diversity, but only complete segment sequences (within 50 base pairs of each end) were included in the construction of phylogenetic trees. A total of twenty virus sequences from other wild bird hosts were included to represent the American and Eurasian avian clades, and were selected from various host species, years and subtypes. Within these broad geographic divisions, American sequences were selected from regions where influenza is regularly isolated across North America and included viruses from Alaska, Alberta, Minnesota, Delaware Bay and New York. Similarly, the selected Eurasian sequences were isolated in the Netherlands, Russia, China and Japan (Table S3). The H13 and H16 phylogenetic trees were constructed using all available H13 and H16 sequences, including those isolated from wild birds other than gulls.

Complete sequences were aligned using ClustalW version 1.4 and the resulting alignments used to construct neighbour-joining trees [58] with 10000 bootstrap replicates [59], all done within MEGA 4.1 [60]. Geographic reassortment was determined by both phylogenetic and BLAST analyses. A geographic reassortant was identified when a segment sequence from a virus isolated in one geographic region fell within a group of viruses detected in a different geographic region.

Genome constellations of the PB2, PB1, PA, NP, M and NS segments of American gull viruses were constructed. Segments were allocated into either a Eurasian clade (reassortant) or an American clade. For the NP, M and NS segments, where gull clades were well supported, segments were further classified into avian or gull clades. Finally, if the segment was allocated into a Eurasian clade, it was classified as a singleton event if it was most similar to a Eurasian virus within that clade, or not if it was most similar to another American virus within a larger Eurasian clade.

## Supporting Information

Figure S1 **Neighbour-joining tree of PB2 sequences.** Grey and blue indicate gull viruses isolated in Eurasia and America, respectively. Red and yellow indicate viruses isolated from other wild bird hosts in Eurasia and America, respectively. Group 1 and group 2 viruses are outlined in Table S6. The scale bar indicates the number of substitutions per site. Bootstrap values are provided as percentages based on 10000 replicates for selected major branch points. The radial tree is presented in Figure 3 of the main text.
(TIF)

Figure S2 **Neighbour-joining tree of PB1 sequences.** Grey and blue indicate gull viruses isolated in Eurasia and America, respectively. Red and yellow indicate viruses isolated from other wild bird hosts in Eurasia and America, respectively. Group 1 and group 2 viruses are outlined in Table S6. The scale bar indicates the number of substitutions per site. Bootstrap values are provided as percentages based on 10000 replicates for selected major branch points. The radial tree is presented in Figure 3 of the main text.
(TIF)

Figure S3 **Neighbour-joining tree of PA sequences.** Grey and blue indicate gull viruses isolated in Eurasia and America, respectively. Red and yellow indicate viruses isolated from other wild bird hosts in Eurasia and America, respectively. Group 1 and group 2 viruses are outlined in Table S6. The scale bar indicates the number of substitutions per site. Bootstrap values are provided as percentages based on 10000 replicates for selected major branch points. The radial tree is presented in Figure 3 of the main text.
(TIF)

Figure S4 **Neighbour-joining tree of NP sequences.** Grey and blue indicate gull viruses isolated in Eurasia and America, respectively. Red and yellow indicate viruses isolated from other wild bird hosts in Eurasia and America, respectively. Group 1 and group 2 viruses are outlined in Table S6. The scale bar indicates the number of substitutions per site. Bootstrap values are provided as percentages based on 10000 replicates for selected major branch points. The radial tree is presented in Figure 3 of the main text.
(TIF)

Figure S5 **Neighbour-joining tree of M sequences.** Grey and blue indicate gull viruses isolated in Eurasia and America, respectively. Red and yellow indicate viruses isolated from other wild bird hosts in Eurasia and America, respectively. Group 1 and group 2 viruses are outlined in Table S6. The scale bar indicates the number of substitutions per site. Bootstrap values are provided as percentages based on 10000 replicates for selected major branch points. The radial tree is presented in Figure 3 of the main text.
(TIF)

Figure S6 **Neighbour-joining tree of NS sequences.** Grey and blue indicate gull viruses isolated in Eurasia and America, respectively. Red and yellow indicate viruses isolated from other wild bird hosts in Eurasia and America, respectively. Branches delineating alleles A and B are identified. Group 1 and group 2 viruses are outlined in Table S6. The scale bar indicates the number of substitutions per site. Bootstrap values are provided as percentages based on 10000 replicates for selected major branch points. The radial tree is presented in Figure 3 of the main text.
(TIF)

Figure S7 **Phylogenetic tree of H2 HA sequences from gulls.** The neighbour-joining tree contains all gull H2 sequences, in addition to reference sequences from other avian hosts. Viruses shaded in grey are those in clade 1 of Figure 3 and Table S6.
(TIF)

Figure S8 **Neighbour-joining trees of the most common NA subtypes of gull viruses.** A. Complete available gull virus N3 sequences. B. Complete available gull virus N6 sequences. The scale bar indicates the number of substitutions per site. Bootstrap values are provided as percentages based upon 10000 replicates.
(TIF)

Table S1 **Primers used for whole genome sequencing of viruses isolated in Alaska.**
(DOC)

Table S2 **Accession numbers of gull virus sequences available in GenBank as of September 2010.**
(DOC)

Table S3 **Reference sequences used to represent the American and Eurasian avian clades for the PB2, PB1, PA, NP, M, and NS segments.**
(DOC)

Table S4 **Availability of sequence information in the GenBank and Influenza Resource Databases for AIV from gulls.**
(DOC)

Table S5 **Hemagglutinin and neuraminidase subtype combination frequency in viruses isolated from gull species globally.**
(DOC)

Table S6 **Identification information for AIV from the Delaware Bay gull community from 1986–1989, which form two distinct clades based on phylogenetic analyses of the PB2, PB1, PA, NP, M, and NS segments.**
(DOC)
