# Supplementary figures and images for: Extensive Geographic Mosaicism in Avian Influenza Viruses from Gulls in the Northern Hemisphere
Source: PLoS One. 2011 Jun 15;6(6):e20664. doi: 10.1371/journal.pone.0020664 (PMC3115932; doi:10.1371/journal.pone.0020664)

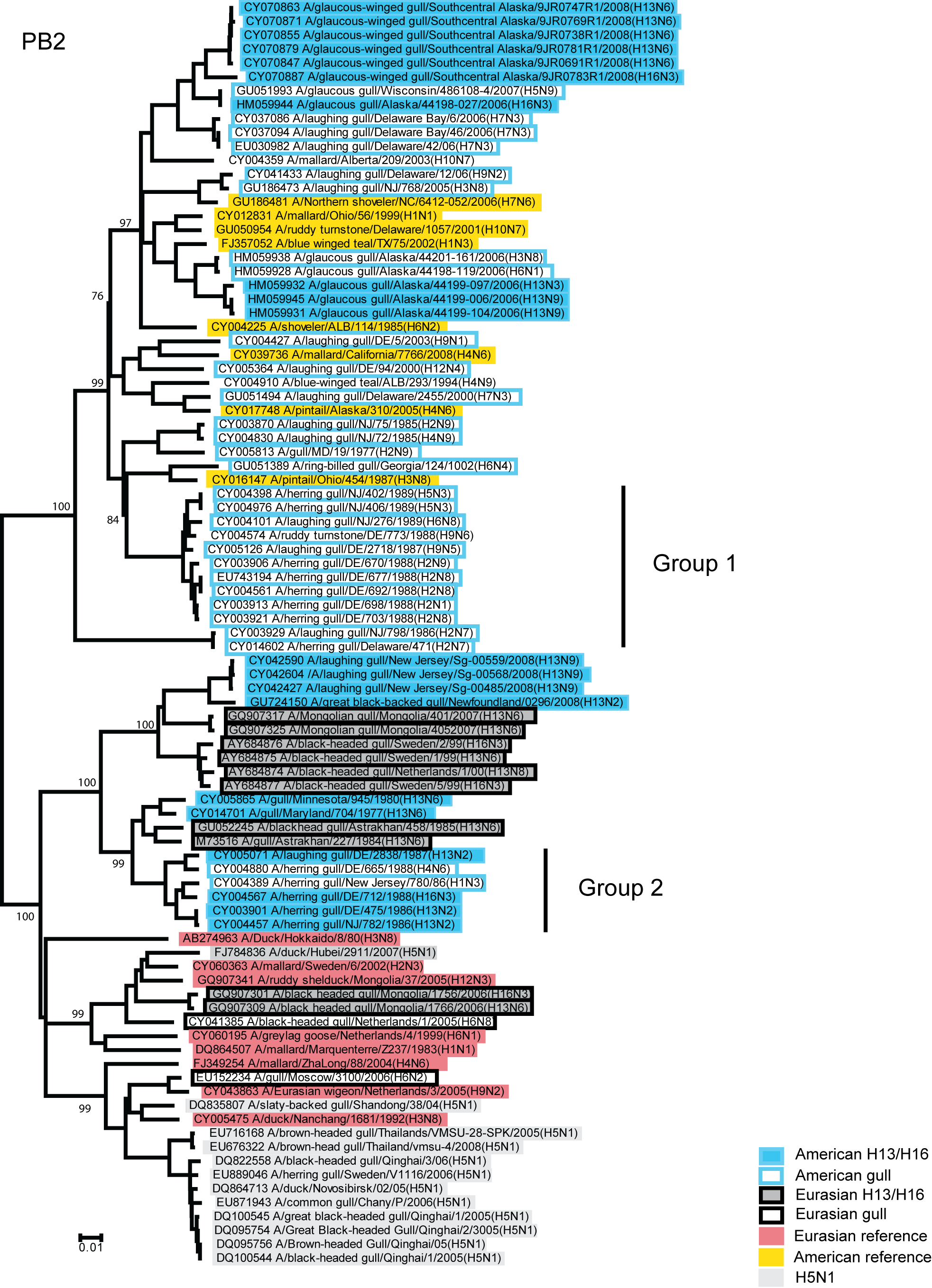

Supplement: Figure S1 — Neighbour-joining tree of PB2 sequences. Grey and blue indicate gull viruses isolated in Eurasia and America, respectively. Red and yellow indicate viruses isolated from other wild bird hosts in Eurasia and America, respectively. Group 1 and group 2 viruses are outlined in Table S6. The scale bar indicates the number of substitutions per site. Bootstrap values are provided as percentages based on 10000 replicates for selected major branch points. The radial tree is presented in Figure 3 of the main text. (TIF) [file pone.0020664.s001.tif]

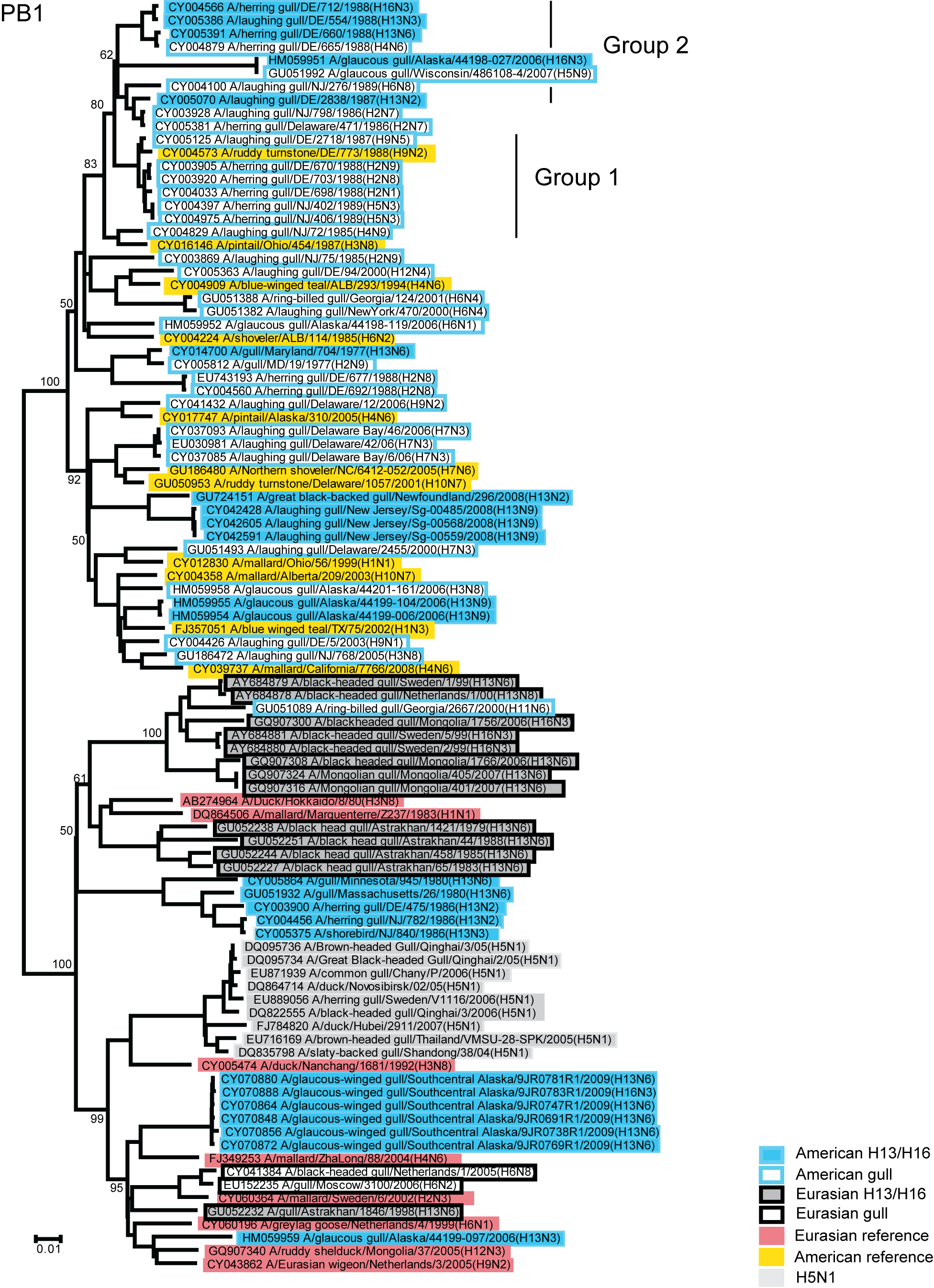

Supplement: Figure S2 — Neighbour-joining tree of PB1 sequences. Grey and blue indicate gull viruses isolated in Eurasia and America, respectively. Red and yellow indicate viruses isolated from other wild bird hosts in Eurasia and America, respectively. Group 1 and group 2 viruses are outlined in Table S6. The scale bar indicates the number of substitutions per site. Bootstrap values are provided as percentages based on 10000 replicates for selected major branch points. The radial tree is presented in Figure 3 of the main text. (TIF) [file pone.0020664.s002.tif]

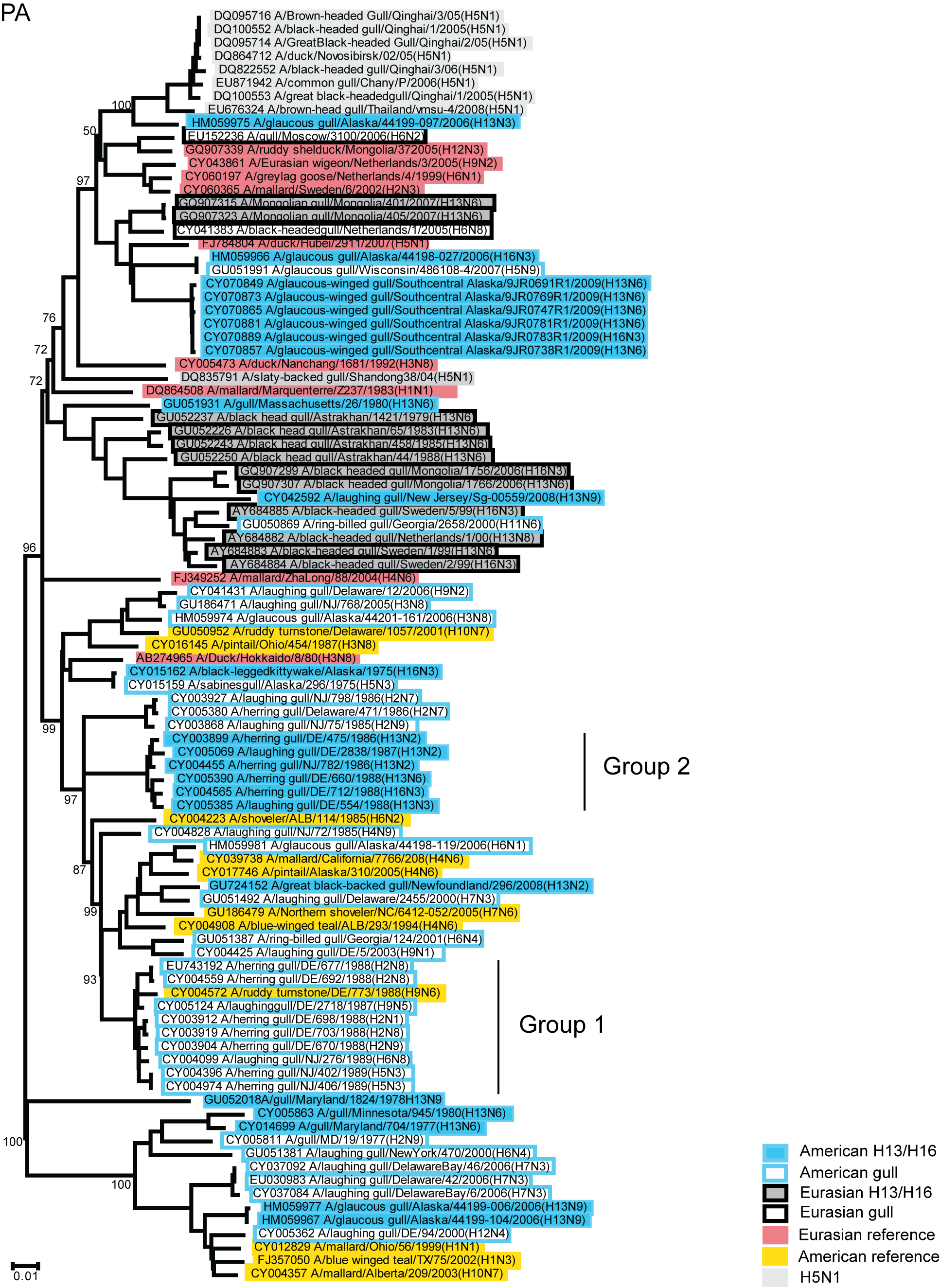

Supplement: Figure S3 — Neighbour-joining tree of PA sequences. Grey and blue indicate gull viruses isolated in Eurasia and America, respectively. Red and yellow indicate viruses isolated from other wild bird hosts in Eurasia and America, respectively. Group 1 and group 2 viruses are outlined in Table S6. The scale bar indicates the number of substitutions per site. Bootstrap values are provided as percentages based on 10000 replicates for selected major branch points. The radial tree is presented in Figure 3 of the main text. (TIF) [file pone.0020664.s003.tif]

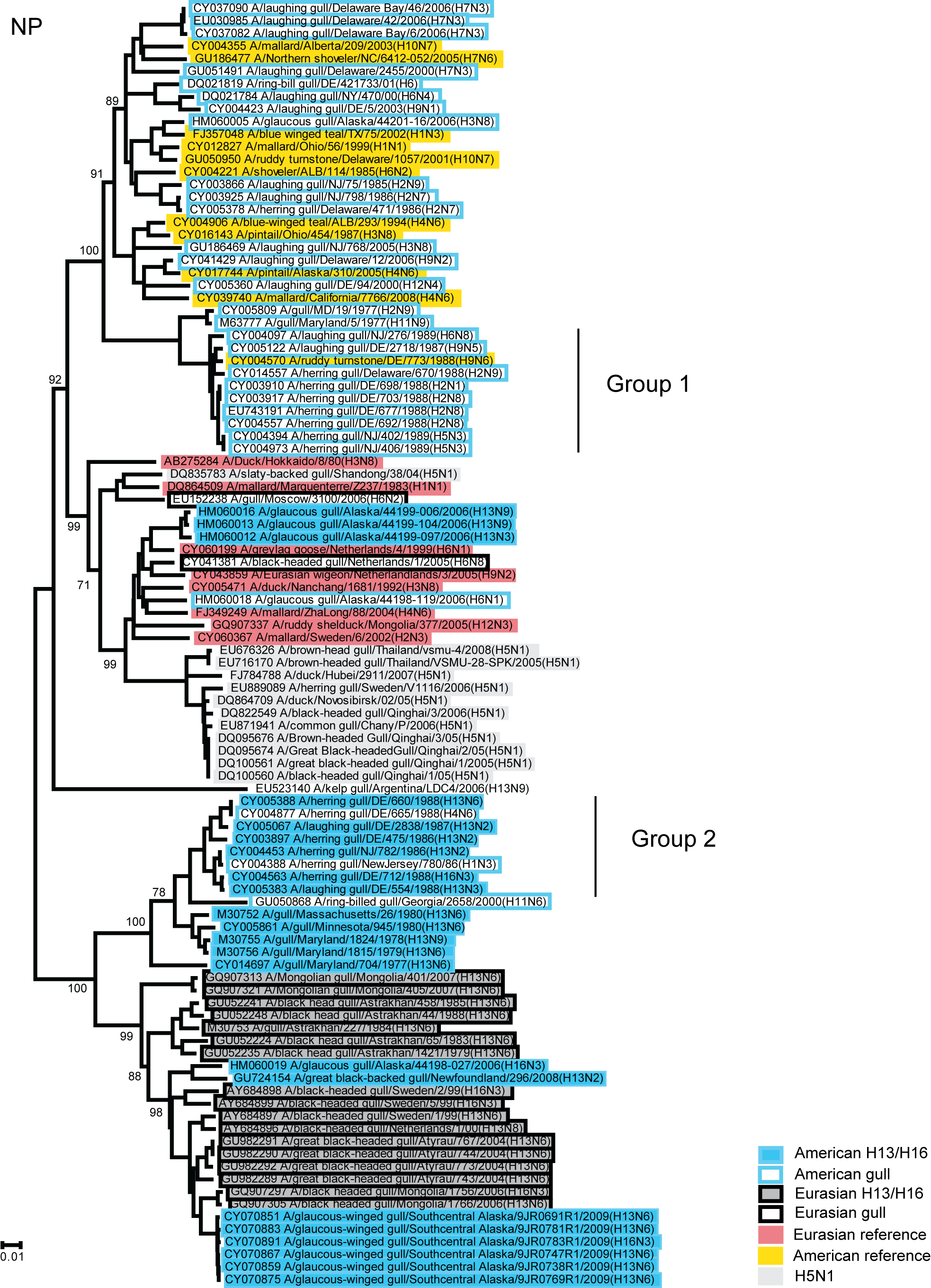

Supplement: Figure S4 — Neighbour-joining tree of NP sequences. Grey and blue indicate gull viruses isolated in Eurasia and America, respectively. Red and yellow indicate viruses isolated from other wild bird hosts in Eurasia and America, respectively. Group 1 and group 2 viruses are outlined in Table S6. The scale bar indicates the number of substitutions per site. Bootstrap values are provided as percentages based on 10000 replicates for selected major branch points. The radial tree is presented in Figure 3 of the main text. (TIF) [file pone.0020664.s004.tif]

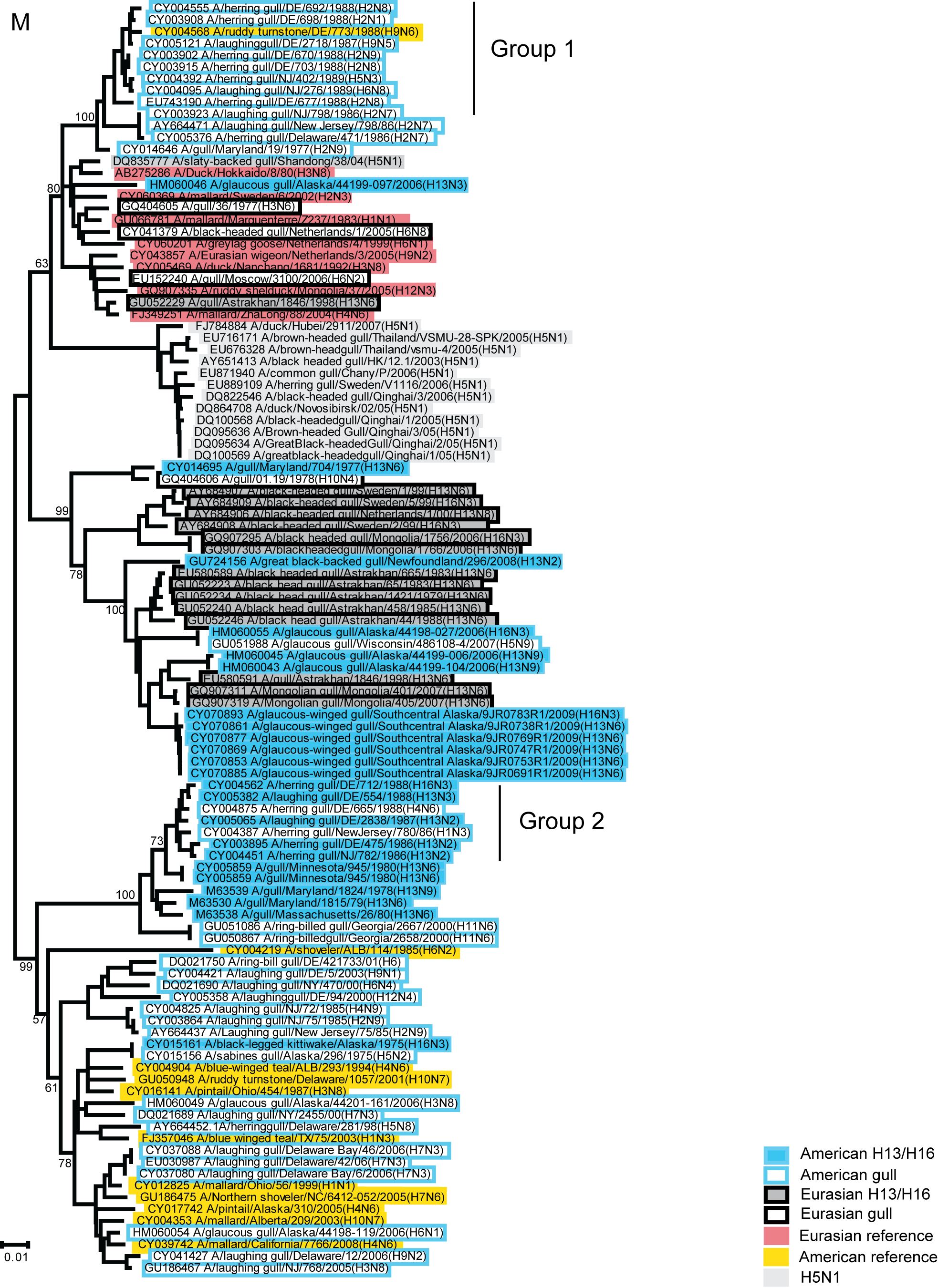

Supplement: Figure S5 — Neighbour-joining tree of M sequences. Grey and blue indicate gull viruses isolated in Eurasia and America, respectively. Red and yellow indicate viruses isolated from other wild bird hosts in Eurasia and America, respectively. Group 1 and group 2 viruses are outlined in Table S6. The scale bar indicates the number of substitutions per site. Bootstrap values are provided as percentages based on 10000 replicates for selected major branch points. The radial tree is presented in Figure 3 of the main text. (TIF) [file pone.0020664.s005.tif]

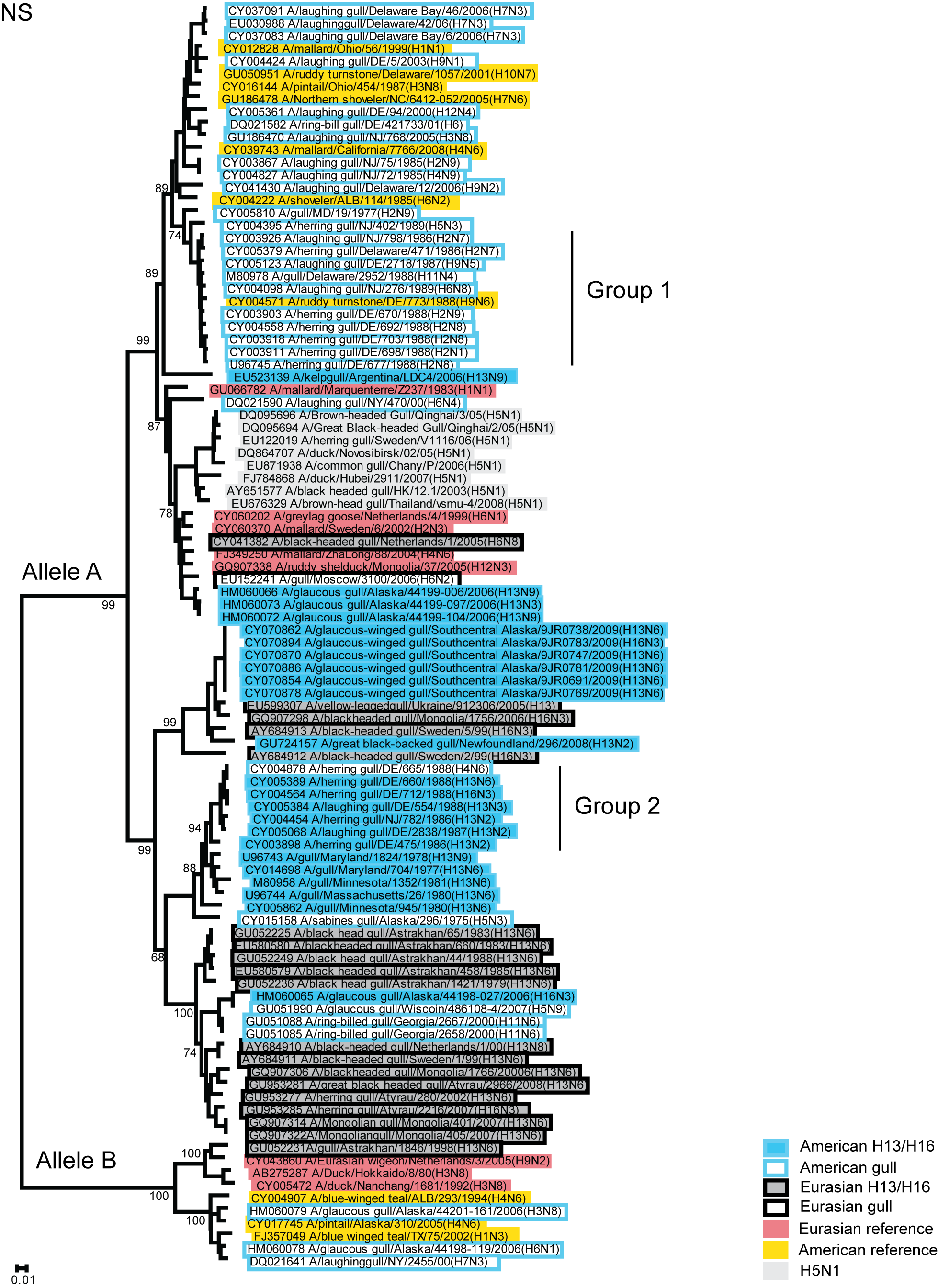

Supplement: Figure S6 — Neighbour-joining tree of NS sequences. Grey and blue indicate gull viruses isolated in Eurasia and America, respectively. Red and yellow indicate viruses isolated from other wild bird hosts in Eurasia and America, respectively. Branches delineating alleles A and B are identified. Group 1 and group 2 viruses are outlined in Table S6. The scale bar indicates the number of substitutions per site. Bootstrap values are provided as percentages based on 10000 replicates for selected major branch points. The radial tree is presented in Figure 3 of the main text. (TIF) [file pone.0020664.s006.tif]

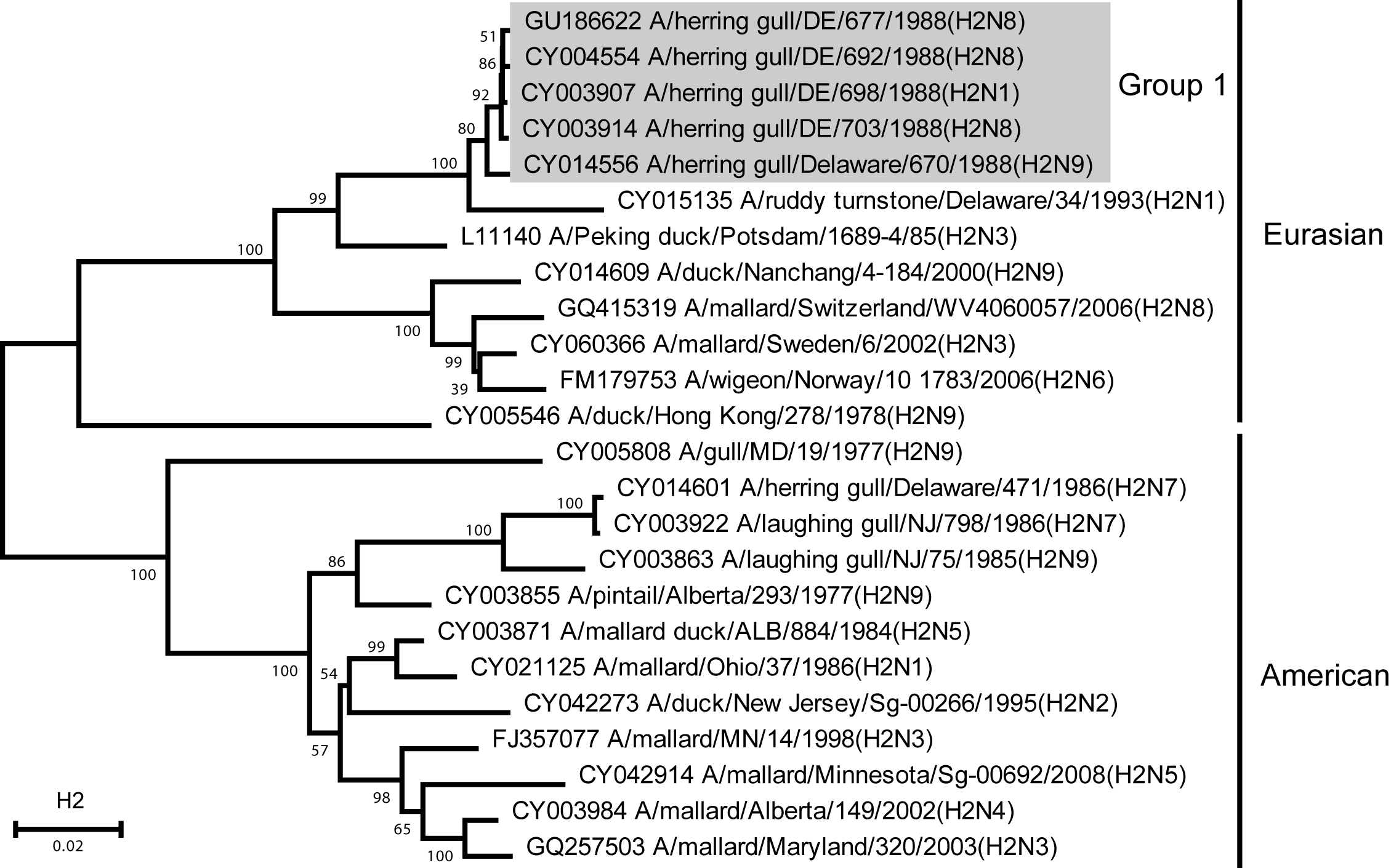

Supplement: Figure S7 — Phylogenetic tree of H2 HA sequences from gulls. The neighbour-joining tree contains all gull H2 sequences, in addition to reference sequences from other avian hosts. Viruses shaded in grey are those in clade 1 of Figure 3 and Table S6. (TIF) [file pone.0020664.s007.tif]

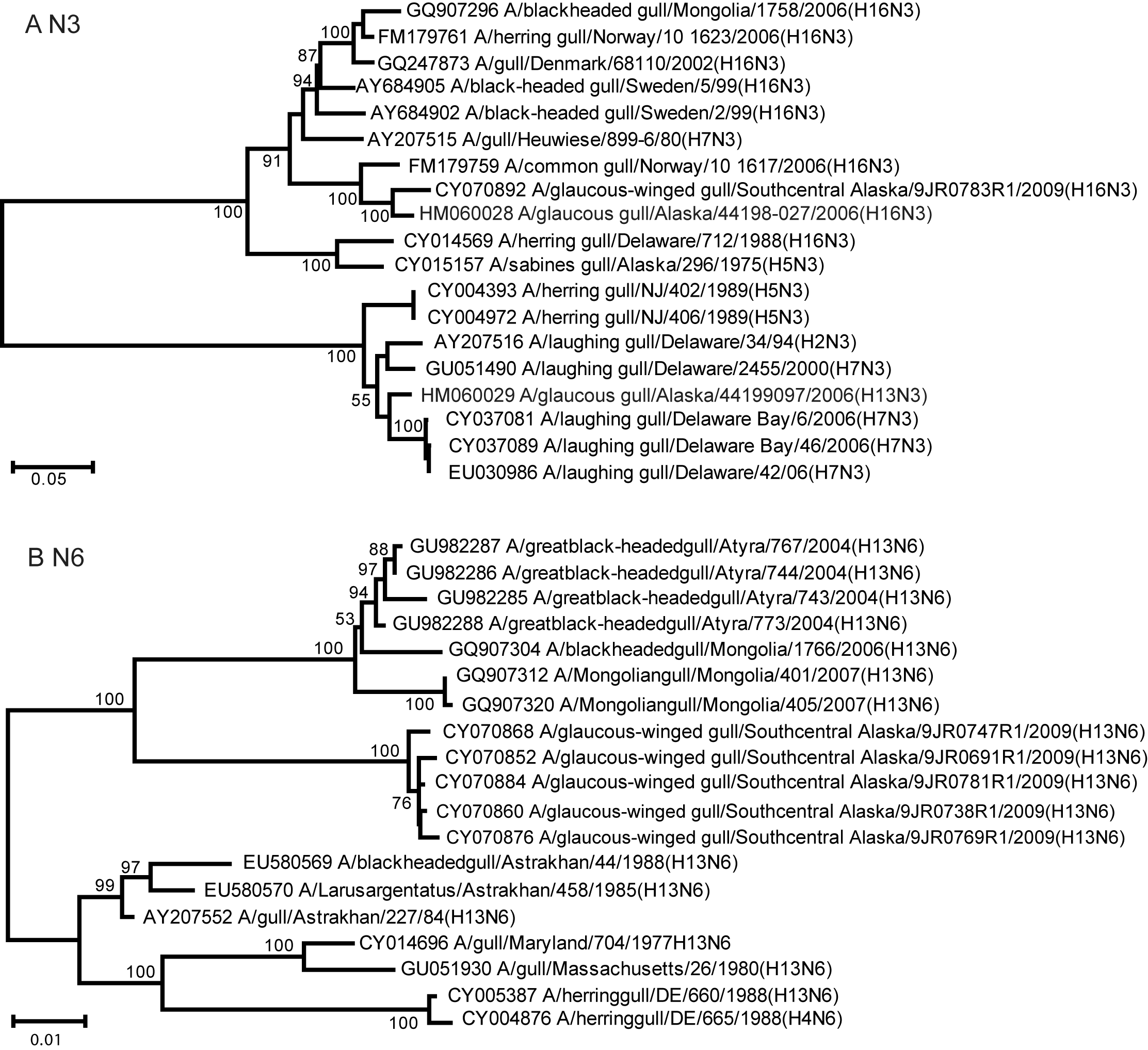

Supplement: Figure S8 — Neighbour-joining trees of the most common NA subtypes of gull viruses. A. Complete available gull virus N3 sequences. B. Complete available gull virus N6 sequences. The scale bar indicates the number of substitutions per site. Bootstrap values are provided as percentages based upon 10000 replicates. (TIF) [file pone.0020664.s008.tif]
