# Supplementary material for: Extensive Geographic Mosaicism in Avian Influenza Viruses from Gulls in the Northern Hemisphere
Source: PLoS One. 2011 Jun 15;6(6):e20664. doi: 10.1371/journal.pone.0020664 (PMC3115932; doi:10.1371/journal.pone.0020664)
Supplement: Table S1 — Primers used for whole genome sequencing of viruses isolated in Alaska. (DOC) [file pone.0020664.s009.doc]

**Table S1.** Primers used for whole genome sequencing of viruses isolated in Alaska.

| Gene Region | Name | Primer | PCR Conditions^a^ | Source |
| --- | --- | --- | --- | --- |
| PB2.1 | Bm-PB2-1 | TATTGGTCTCAGGGAGCGAAAGCAGGTC | 2 | [1] |
|  | PB2-1250R | TCYTCYTGTGARAAYACCAT |  | [2] |
| PB2.2 | PB2-1105F | TAYGARGARTTCACAATGGT | 1 | [2] |
|  | B010 PB2-1962R | ATTCCKGAGCCTCTCACATTCACAG |  | [3] |
| PB2.3 | B022 PB2.2-1814F | TGAGGACACTGTTCCAGCAGATG | 1 | [3] |
|  | AVAKPB222R | CAAGGTCGTTTTTAAACAATTCG |  | [4] |
| PB1.1 | Bm-PB1-1 | TATTCGTCTCAGGGAGCGAAAGCAGGCA | 2 | [1] |
|  | A006R PB1-621R | CATTTTCTTGGTCATGTTG |  | [3] |
| PB1.2 | A014F PB1-533F | TAGATTTTCTCAAGGATGTGATGGA | 2 | [3] |
|  | PB1-1262R | TTRAACATGCCCATCATCAT |  | [2] |
| PB1.3 | PB1-1124F | ARATACCNGCAGARATGCT | 2 | [2] |
|  | A012 PB1-1964R | GGCATTACCACAGCATTGTT |  | [3] |
| PB1.4 | PB1-1564 | GGAGCTGCCCAGCTTTGGAG | 2 | [3] |
|  | Bm-PB1-2341R | ATATCGTCTCGTATTAGTAGAAACAAGGCATTT |  | [1] |
| PA.1 | Bm-PA-1 | TATTCGTCTCAGGGAGCGAAAGCAGGTAC | 1 | [1] |
|  | C006 PA607R | CGGATTGACGAAAGGAGTCCC |  | [3] |
| PA2 | C005 PA587F | GGGAYTCCTTTCGTCAGTCCG | 1 | [3] |
|  | PA-1498R | TNGTYCTRCAYTTGCTTATCAT |  | [2] |
| PA.3 | PA-747F | CATTGAGGGCAAGCTTTC | 1 | [2] |
|  | C011 PA1919R | GARCCTTCYTCCACYCCYTTRGG |  | [3] |
| PA4 | PA1543 | GGARTTYTCYCTYACWGAYCC | 1 | [3] |
|  | Bm-PA-2233R | ATATCGTCTCGTATTAGTAGAAACAAGGTACTT |  | [1] |
| NP.1 | SZANPF | CTCGAGAGCAAAAGCAGGGT | 1 | [5] |
|  | E010 NP-734R | AATTTCCCTTTGAGGATGTTGCACATTC |  | [3] |
| NP.2 | E003 NP-517F | GGAATGGAYCCCAGGATGTGCTC | 1 | [3] |
|  | SZANPR | AGTAGAAACAAGGGTATTTTTC |  | [5] |
| M | SZAMF | CTCGAGCAAAAGCAGGTAGAT | 1 | [5] |
|  | SZAMR | ATGAGAAACAAGGTAGTTTTT |  | [5] |
| NS | SZANSF | AGCAAAAGCAGGGTGACAAA | 1 | [5] |
|  | SZANSR | ATGAGAAACAAGGGTGTTTTTT |  | [5] |
| H13.1 | HA_av_H13_M13_1F | TGTAAAACGACGGCCAGTAGCAAAAGCAGGGGA | 1 | [6] |
|  | HA_av_H13_M13_783R | CAGGAAACAGCTATGACCCCARTAAATYTTCATCCARCTC |  | [6] |
| H13.2 | HA_av_H13_M13_579F | TGTAAAACGACGGCCAGTGACYTACAACAAYACHACKGGHAG | 1 | [6] |
|  | HA_av_H13_M13_1195R | CAGGAAACAGCTATGACCGTGTYGAYTCTTTRTCTGCAGC |  | [6] |
| H13.3 | HA_av_H13_M13_621F | TGTAAAACGACGGCCAGTGGGDATACAYCAYCCWGT | 1 | [6] |
|  | HA_av_H13_M13_1265R | CAGGAAACAGCTATGACCGARTCATARTTYCCRTTCATTTTDTC |  | [6] |
| H13.4 | HA_av_H13_M13_1088F | TGTAAAACGACGGCCAGTTTGCAGGVTTCATAGAAGGWGG | 1 | [6] |
|  | HA_av_H13_M13_1770R | CAGGAAACAGCTATGACCAGTAGAAACAAGGGTRTTT |  | [6] |
| H16.1 | HA_av_H16_M13_1F | TGTAAAACGACGGCCAGTAGCAAAAGCAGGGGATA | 1 | [6] |
|  | HA_av_H16_M13_783R | CAGGAAACAGCTATGACCATBARGTGCCAATADAKTTTCATCC |  | [6] |
| H16.2 | HA_av_H16_M13_591F | TGTAAAACGACGGCCAGTCAGGCAGAGATGTTYTAG | 1 | [6] |
|  | HA_av_H16_M13_1227R | CAGGAAACAGCTATGACCATATTRTTWATYTTTGTTGTTATYTC |  | [6] |
| H16.3 | HA_av_H16_M13_620F | TGTAAAACGACGGCCAGTATTCACCAYCCDGAYACAG | 1 | [6] |
|  | HA_av_H16_M13_1302R | CAGGAAACAGCTATGACCAKCATGTTGATYCTCTTTTCYA |  | [6] |
| H16.4 | HA_av_H16_M13_1085F | TGTAAAACGACGGCCAGTGGMTTYATAGAAGGAGGRTGG | 1 | [6] |
|  | HA_av_H16_M13_1761R | CAGGAAACAGCTATGACCAGTAGAAACAAGGGTRTTT |  | [6] |
| NA.1 | NAF M | TATTACGCGTCGAGGGAGCAAAAGCAGGAGT | 1 | [7] |
| NA.2 | NAR K | ATATGGCGCCGTATTAGTAGAAACAAGGAGTTTTTT | 1 | [7] |
| N1 | N1.1 | GAACAGGCAGTTGTGGTC | 3 | [8] |
|  | N1.2 | TYAGTTCTGGATGCTGGA |  | [8] |
| N2 | N2.1 | TCCGTTTCATTTGGGAAC | 3 | [8] |
|  | N2.2 | CTGACAATGGRCTAATGTG |  | [8] |
| N3 | N3.1 | ATCATGTGAYTCYCCAAG | 3 | [8] |
|  | N3.2 | TCCCGATCCAGGTTCAT |  | [8] |
| N4 | N4.1 | ATGTGCATGCAACAGGGTTC | 3 | [8] |
|  | N4.2 | CTGTTGTCTCYCCTCTAATGC |  | [8] |
| N5 | N5.1 | AYCCTGCAACACCACTGAG | 3 | [8] |
|  | N5.2 | TCTCTTTCATTTGTCACCAT |  | [8] |
| N6 | N6.1 | AACCGGAGGGAGCCCAGATC | 3 | [8] |
|  | N6.2 | TCCCAATCGCTCYTTGGATC |  | [8] |
| N7 | N7.1 | ATGYTGAARATACCYAATGC | 3 | [8] |
|  | N7.2 | ARGAACCRGAACCAACTG |  | [8] |
| N8 | N8.1 | ACAGTCRTTAGGGAATAC | 3 | [8] |
|  | N8.2 | TACACATTGGGTGATG |  | [8] |
| N9 | N9.1 | TGTAATGACCCTTATCCAGG | 3 | [8] |
|  | N9.2 | GTTCCATTGTCCAAGGAATTC |  | [8] |

a Reactions contained 0.5 μl cDNA, 0.3 mM MgCl2, 0.2 mM each dNTP (New England

Biolabs), 0.2 mM of each primer, and 0.5 U Platinum Taq DNA Polymerase (Invitrogen)

in a final volume of 25 μl 1x PCR Buffer (Invitrogen).

1. Thermocycler conditions: 2 min at 94°C, followed by 40 cycles of 94°C for 30s, 55°C

for 30s, 72°C for 2min, followed by 72°C for 10min.

2. Thermocycler conditions: 2 min at 94°C, followed by 40 cycles of 94°C for 30s, 52°C

for 30s, 72°C for 2min, followed by 72°C for 10min.

3. Thermocycler conditions: 2 min at 94°C, followed by 40 cycles of 94°C for 40s, 60°C

for 40s, 72°C for 40s, followed by 72°C for 10min.

References

1. Hoffmann E, Stech J, Y. G, Webster RG, Perez DR (2001) Universal primer set for the full-length amplification of all influenza A viruses. Arch Virol 146: 2275-2289.

2. Li OTW, Barr I, Leung CYH, Chen H, Guan Y, et al. (2007) Reliable universal RT-PCR assays for studying influenza polymerase subunit gene sequences from all 16 hemagglutinin subtypes. J Virol Methods 142: 218-222.

3. Obenauer JC, Denson J, Mehta PK, Su X, Mukatira S, et al. (2006) Large-scale sequence analysis of avian influenza isolates. Science 311: 1576 - 1580.

4. Koehler AV, Pearce JM, Flint PL, Franson JC, Ip HS (2008) Genetic evidence of interncontinental movement of avian influenza in a migratory bird: the Northern Pintail (*Anas acuta*). Mol Ecol 17: 4754-4762.

5. Zou S (1999) A practical approach to genetic screening for influenza virus variants. J Clin Microbiol 35: 2623-2627.

6. Dugan VG, Chen R, Spiro DJ, Sengamalay N, Zaborsky J, et al. (2008) The evolutionary genetics and emergence of avian influenza A viruses in wild birds. PLoS Pathog 4: e1000076.

7. Bragstad K, Jorgensen PH, Handberg KJ, Mellergaard S, Corbet S, et al. (2005) New avian influenza A subtype combination H7N5 identified in Danish Mallard ducks. Virus Res 109: 181-190.

8. Qiu B-F, Liu W-J, Peng D-X, Hu S-L, Tang Y-H, et al. (2009) A reverse-transcription-PCR for subtyping of the neuraminidase of avian influenza viruses. J Virol Methods 155: 193-198.
