# Supplementary material for: Extensive Geographic Mosaicism in Avian Influenza Viruses from Gulls in the Northern Hemisphere
Source: PLoS One. 2011 Jun 15;6(6):e20664. doi: 10.1371/journal.pone.0020664 (PMC3115932; doi:10.1371/journal.pone.0020664)
Supplement: Table S2 — Accession numbers of gull virus sequences available in GenBank as of September 2010. (DOC) [file pone.0020664.s010.doc]

**Table S2.** Accession numbers of gull virus sequences available in GenBank as of September 2010.

| Virus Name | Segment | | | | | | | | | |
| --- | --- | --- | --- | --- | --- | --- | --- | --- | --- | --- |
|  | PB2 | PB1 | PA | HA | | NP | NA | | M | NS |
| A/gull/01.19/1978(H10N4) |  |  |  |  | |  |  | | GQ404606 |  |
| A/gull/Delaware/2952/1988(H11N4) |  |  |  |  | |  | AY207545 | |  | M80978 |
| A/ring-billed gull/Georgia/2658/2000(H11N6) |  |  |  |  | |  |  | | GU051386 | GU051085 |
| A/ring-billed gull/Georgia/2667/2000(H11N6) |  | GU051089 | GU050869 |  | | GU050868 |  | | GU050867 | GU051088 |
| A/gull/Maryland/5/1977(H11N9) |  |  |  |  | | M63777 |  | |  |  |
| A/laughing gull/DE/94/2000(H12N4) | CY005364 | CY005363 | CY005362 | CY014598 | | CY005360 | CY005359.1 | | CY005358 | CY005361 |
| A/herring gull/Germany/wv1136KRK/03(H13) |  |  |  | AM087221 | |  |  | |  |  |
| A/Larus ridibundus/Germany/R2064/2006(H13) |  |  |  | AM922164 | |  |  | |  |  |
| A/yellow-legged gull/Ukraine/912306/2005(H13) |  |  |  | EU599306 | |  |  | |  | EU599307 |
| A/Great Black-backed Gull/Newfoundland/296/2008(H13N2) | GU724150 | GU724151 | GU724152 | GU724153 | | GU724154 | GU724155 | | GU724156 | GU724157 |
| A/great black-headed gull/Astrakn/1420/79(H13N2) |  |  |  | EU293858 | |  |  | |  |  |
| A/great black-headed gull/Astrakn/591/82(H13N2) |  |  |  | EU293860 | |  |  | |  |  |
| A/great black-headed gull/Gurjev/76/83(H13N2) |  |  |  | EU293861 | |  |  | |  |  |
| A/gull/Astrakn/176/1986(H13N2) |  |  |  | EU835899 | |  |  | |  |  |
| A/herring gull/DE/475/1986(H13N2) | CY003901 | CY003900 | CY003899 | CY005914 | | CY003897 | CY003896 | | CY003895 | CY003989 |
| A/herring gull/NJ/782/1986(H13N2) | CY004457 | CY004456 | CY004455 | CY005932 | | CY004453 | CY004452 | | CY004451 | CY004454 |
| A/Larus ichthyaetus/Astrakn/75/1983(H13N2) |  |  |  | EU564107 | |  |  | |  |  |
| A/laughing gull/DE/2838/1987(H13N2) | CY005071 | CY005070 | CY005069 | CY005979 | | CY005067 | CY005066 | | CY005065 | CY005068 |
| A/laughing gull/DE/554/1988(H13N3) |  | CY005386 | CY005385 |  | | CY005383 |  | | CY005382 | CY005384 |
| A/laughing gull/New Jersey/171/92(H13N5) |  |  |  | AF310992 | |  |  | |  |  |
| A/black-headed gull/Mongolia/1766/2006(H13N6) | GQ907309 | GQ907308 | GQ907307 | GQ907302 | | GQ907305 | GQ907304 | | GQ907303 | GQ907306 |
| A/black-headed gull/Sweden/1/99(H13N6) | AY684875 | AY684879 | AY684883 | AY684887 | | AY684897 | AY684901 | | AY684907 | AY684911 |
| A/black headed gull/Astrakan/1421/1979(H13N6) or A/great black-headed gull/Astrakan/1421/1979(H13N6) | GU052239 | GU052238 | GU052237 | EU293859 | | GU052235 |  | | GU052234 | GU052236 |
| A/Larus ichthyaetus/Astrakn/44/1988(H13N6) | GU052252 | GU052251 | GU052250 | EU564115 | | GU052248 | GU052247 | | GU052246 | GU052249 |
| A/black headed gull/Astrakhan/458/1985(H13N6) | GU052245 | GU052244 | GU052237 |  | | GU052241 |  | | GU052240 | EU580579/GU052242 |
| A/black headed gull/Astrakhan/65/1983(H13N6) | GU052228 | GU052227 | GU052237 | EU580577 | | GU052224 |  | | GU052223 | GU052225 |
| A/gull/Astrakn/1314/1979(H13N2) |  |  |  | EU835898 | |  |  | |  |  |
| A/gull/Astrakhan/1818/1998(H13N6) |  |  |  | EU835900 | |  |  | |  |  |
| A/gull/Astrakhan/1846/1998(H13N6) | GU052233 | GU052234 |  | EU580576 | | GU052230 |  | | GU052229 | GU052231 |
| A/gull/Astrakhan/226/1984(H13N6) |  |  |  | EU835895 | |  |  | |  |  |
| A/gull/Astrakhan/227/1984(H13N6) or A/black head gull/Astrakhan/227/1984(H13N6) | M73516 |  |  | M26089 | | M30753 | AY207552 |  | |  |
| A/gull/Astrakhan/3483/2002(H13N6) |  |  |  | EU835897 | |  |  |  | |  |
| A/gull/Astrakhan/998/1990(H13N6) |  |  |  | EU835896 | |  |  |  | |  |
| A/glaucous-winged gull/Southcentral_Alaska/ 9JR0691R1/2009(H13N6) | CY070847 | CY070848 | CY070849 | CY070850 | | CY070851 | CY070852 | | CY070853 | CY070854 |
| A/glaucous-winged gull/Southcentral_Alaska/ 9JR0738R1/2009(H13N6) | CY070855 | CY070856 | CY070857 | CY070858 | | CY070859 | CY070860 | | CY070861 | CY070862 |
| A/glaucous-winged gull/Southcentral_Alaska/ 9JR0747R1/2009(H13N6) | CY070863 | CY070864 | CY070865 | CY070866 | | CY070867 | CY070868 | | CY070869 | CY070870 |
| A/gull/Stralsund/Wv1136-40/03(H13N6) |  |  |  | AM922163 | |  |  | |  |  |
| A/herring gull/Astrakn/458/85(H13N6) |  |  |  | EU293863 | |  | EU580570 | |  |  |
| A/herring gull/Astrakn/479/85(H13N6) |  |  |  | EU293862 | |  | EU580570 | |  |  |
| A/glaucous-winged gull/Southcentral_Alaska/ 9JR0769R1/2009(H13N6) | CY070871 | CY070872 | CY070873 | CY070874 | | CY070875 | CY070876 | | CY070877 | CY070878 |
| A/herring gull/Norway/10_2336/2006(H13N6) |  |  |  | FM179758 | | EU564106 |  | |  |  |
| A/Larus ichthyaetus/Astrakn/10/1988(H13N6) |  |  |  | EU564106 | |  |  | |  |  |
| A/Larus minutus/Astrakn/3357/2002(H13N6) |  |  |  | EU564108 | |  |  | |  |  |
| A/Mongolian gull/Mongolia/401/2007(H13N6) | GQ907317 | GQ907316 | GQ907315 | GQ907310 | | GQ907313 | GQ907312 | | GQ907311 | GQ907314 |
| A/Mongolian gull/Mongolia/405/2007(H13N6) | GQ907325 | GQ907324 | GQ907323 | GQ907318 | | GQ907321 | GQ907320 | | GQ907319 | GQ907322 |
| A/glaucous-winged gull/SouthcentralAlaska/9JR0781R1/2009(H13N6) | CY070879 | CY070880 | CY070881 | CY070882 | | CY070883 | CY070884 | | CY070885 | CY070886 |
| A/gull/Maryland/704/1977H13N6 | CY014701 | CY014700 | CY014699 | CY014694 | | CY014697 | CY014696 | | CY014695 | CY01498 |
| A/gull/Massachusetts/26/1980(H13N6) |  | GU051932 | GU051931 | K00383 | | M30752 | GU051930 | | M63538 | U96744 |
| A/gull/Minnesota/1352/1981(H13N6) |  |  |  |  | |  |  | |  | M80958 |
| A/herring gull/DE/660/1988(H13N6) |  | CY005391 | CY005390 | CY014603 | | CY005388 | CY005387 |  | | CY005389 |
| A/great black headed gull/Atyrau/2966/2008/H13N6 |  |  |  | GU953282 | | GU953280 | GU953287 | | GU953279 | GU953281 |
| A/herringgull/Atyrau/280/2002/H13N6 |  |  |  | GU953278 | | GU953276 |  | | GU953275 | GU953277 |
| A/great black headed gull/Atyrau/742/2004/H13N6 |  |  |  |  | |  | GU953289 |  | |  |
| A/great black headed gull/Atyrau/773/2004/H13N6 |  |  |  | GU982284 | | GU982292 | GU982288 |  | |  |
| A/great black headed gull/Atyrau/767/2004/H13N6 |  |  |  | GU982283 | | GU982291 | GU982287 |  | |  |
| A/great black headed gull/Atyrau/744/2004/H13N6 |  |  |  | GU982282 | | GU982290 | GU982286 |  | |  |
| A/great black headed gull/Atyrau/743/2004/H13N6 |  |  |  | GU982281 | | GU982289 | GU982285 |  | |  |
| A/black-headed gull/Netherlands/1/00(H13N8) | AY684874 | AY684978 | AY684882 | AY684886 | | AY684896 | AY684900 | | AY684906 | AY684910 |
| A/black-headedgull/Norway/10_1459/2006H13N8 |  |  |  |  | |  | FM179764 | |  |  |
| A/gull/Maryland/1815/1979(H13N9) |  |  |  |  | | M30756 |  | | M63530 |  |
| A/gull/Maryland/1824/1978(H13N9) |  |  | GU052018 |  | | M30755 |  | | M63539 | U96743 |
| A/gull/Minnesota/945/1980(H13N9) | CY005865 | CY005864 | CY005863 | CY014720 | | CY005861 | CY005860 | | CY005859 | CY005862 |
| A/kelp gull/Argentina/LDC4/2006(H13N9) | EU523143 | EU523142 | EU523141 | EU523136 | | EU523140 | EU523137 | | EU523138 | EU523139 |
| A/laughing gull/New Jersey/Sg-00485/2008(H13N9) | CY042427 | CY042428 |  | CY042429 |  | | CY042430 |  | |  |
| A/laughing gull/New Jersey/Sg-00559/2008(H13N9) | CY042590 | CY042591 | CY042592 | CY042593 |  | | CY042594 |  | |  |
| A/laughing gull/New Jersey/Sg-00568/2008(H13N9) | CY042604 | CY042605 | CY042606 | CY042607 |  | | CY042608 |  | |  |
| A/herring gull/Astrakn/267/1982(H14N5) |  |  |  | FJ975075 | |  |  | |  |  |
| A/glaucous-winged gull/Southcentral Alaska/9JR0783R1/2009(H16N3) | CY070887 | CY070888 | CY070889 | CY070890 | | CY070891 | CY070892 | | CY070893 | CY070894 |
| A/black-headed gull/Mongolia/1756/2006(H16N3) | GQ907301 | GQ907300 | GQ907299 | GQ907294 | | GQ907297 | GQ907296 | | GQ907295 | GQ907298 |
| A/black-headed gull/Sweden/2/99(H16N3) | AY684876 | AY684880 | AY684884 | AY684888 | | AY684898 | AY684902 | | AY684908 | AY684912 |
| A/black-headed gull/Turkmanistan/13/76(H16N3) |  |  |  | EU293864 |  | |  | |  |  |
| A/black-headed gull/Sweden/5/99(H16N3) | Ay684877 | AY684881 | AY684885 | AY684891 | AY684899 | | AY684905 | | AY684909 | AY684913 |
| A/common gull/Norway/10_1617/2006(H16N3) |  |  |  | FM179755 |  | | FM179759 |  | |  |
| A/gull/Denmark/68110/2002(H16N3) |  |  |  | GQ247872 |  | | GQ247873 |  | |  |
| A/herring gull/Norway/10_1623/2006(H16N3) |  |  |  | FM179756 |  | | FM179761 |  | |  |
| A/slender-billed gull/Astrakn/28/76(H16N3) |  |  |  | EU293865 |  | |  | |  |  |
| A/black-legged kittiwake/Alaska/295/1975(H16N3) |  |  | CY015162 | CY015160 |  | |  | | CY015161 |  |
| A/herring gull/DE/712/1988(H16N3) | CY004567 | CY004566 | CY004565 | CY005933 | CY004563 | | CY014569 | | CY004562 | CY004564 |
| A/herring gull/Atyrau/2216/2007/H16N3 |  |  |  | GU953286 | GU953284 | |  | | GU953283 | GU953285 |
| A/black-headed gull/Sweden/3/99(H16N3) |  |  |  | AY684889 |  | | AY684903 |  | |  |
| A/black-headed gull/Sweden/4/99(H16N3) |  |  |  | AY684890 |  | | AY684904 |  | |  |
| A/herring gull/NewJersey/780/86(H1N3) | CY004389 |  |  |  | CY004388 | |  | CY004387 | |  |
| A/laughinggull/Delaware/34/94H2N3 |  |  |  |  |  | | AY207516 |  | |  |
| A/herring gull/DE/698/1988(H2N1) | CY003913 | CY004033 | CY003912 | CY003907 | | CY003910 | CY003909 | | CY003908 | CY003911 |
| A/herring gull/Delaware/471/86(H2N7) | CY014602 | CY005381 | CY005380 | CY014601 | | CY005378 | CY005377 | | CY005376 | CY005379 |
| A/laughing gull/NJ/798/1986(H2N7) | CY003929 | CY003928 | CY003927 | CY003922 | | CY003925 | CY003924 | | CY003923 | CY003926 |
| A/herring gull/DE/677/1988(H2N8) | EU743194 | EU743193 | EU743192 | GU186622 | | EU743191 | GU186623 | | EU743190 | U96745 |
| A/herring gull/DE/692/1988(H2N8) | CY004561 | CY004560 | CY004559 | CY004554 | | CY004457 |  | | CY004555 | CY004558 |
| A/herring gull/DE/703/1988(H2N8) | CY003921 | CY003920 | CY003919 | CY003914 | | CY003917 | CY003916 | | CY00915 | CY003918 |
| A/gull/MD/19/1977(H2N9) | EU742651 | EU742650 | EU742649 | EU742644 | | EU742647 | EU742646 | | EU742645 | EU742648 |
| A/herring gull/DE/670/1988(H2N9) | CY003906 | CY003905 | CY003904 | CY014556 | | CY014557 |  | | CY003902 | CY003903 |
| A/laughing gull/NJ/75/1985(H2N9) | CY003870 | CY003869 | CY003868 | CY003863 | | CY003866 | CY004826 | | CY003864 | CY003867 |
| A/gull/36/1977(H3N6) |  |  |  |  | |  |  | | GQ404605 |  |
| A/laughing gull/NJ/768/2005(H3N8) | GU186473 | GU186472 | GU186471 | GU186466 | | GU186469 | GU186468 | | GU186467 | GU186470 |
| A/herring gull/DE/665/1998(H4N6) | CY004880 | CY004879 |  |  | | CY004877 | CY004876 | | CY004875 | CY004878 |
| A/laughing gull/NJ/72/1985(H4N9) | CY004830 | CY004829 | CY004828 |  | |  | CY004826 | | CY004825 | CY004827 |
| A/black-headed gull/HK/12.1/2003/H5N1 | AY651743 | AY651689 | AY651635 | AY651373 | | AY651523 | AY651470 | | AY651413 | AY651577 |
| A/black-headed gull/Qingan/1/2005(H5N1) | DQ100544 | DQ100548 | DQ100552 | DQ100556 | | DQ100560 | DQ100564.1 | | DQ100568 | DQ100572 |
| A/black-headed gull/Qinghan/3/2006(H5N1) | DQ822558 | DQ822555 | DQ822552 | DQ822564 | | DQ822549 | DQ822561 | | DQ822546 | DQ822543 |
| A/black-headed gull/Tyva/115/2009(H5N1) |  |  |  | GQ338084 | |  | GQ338085 | |  |  |
| A/brown-headed gull/Qinghai/03/05(H5N1) | DQ095756 | DQ095736 | DQ095716 | DQ095616 | | DQ095676 | DQ095656 | | DQ95636 | DQ095696 |
| A/brown-headed gull/Qinghai/03/07(H5N1) |  |  |  | FJ602806 | |  | FJ602842 |  | |  |
| A/brown-headed gull/Qinghai/04/07(H5N1) |  |  |  | FJ602807 | |  | FJ602843 |  | |  |
| A/brown-headed gull/Qinghai/06/07(H5N1) |  |  |  | FJ602867 | |  | FJ602845 |  | |  |
| A/brown-headed gull/Thailand/VSMU-28-SPK/2005(H5N1) | EU716168 | EU716169 |  | EF178528 | | EU716170 | EF178529 | | EU716171 |  |
| A/brown-headed gull/Thailand/VSMU-4/2008(H5N1) | EU676322 | EU676323 | EU676324 | EU676325 | |  | EU676327 | | EU676328 | EU676329 |
| A/common gull/Chany/P/2008 (H5N1) | EU871943 | EU871939 | EU871942 | EU871935 | | EU871941 | EU871937 | | EU871940 | EU871938 |
| A/Great Black-headed gull/Qinghai/12/2007(H5N1) |  |  |  | FJ602814 | |  | FJ602851 |  | |  |
| A/Great Black-headed gull/Qinghai/2/2005(H5N1) | DQ09754 | DQ095734 | DQ095714 | DQ095614 | | DQ095674 | DQ095654 | | DQ095634 | DQ095694 |
| A/Great Black-headed gull/Qinghai/2/2007(H5N1) |  |  |  | FJ461726 | |  |  | |  |  |
| A/Great Black-headed gull/Qinghai/8/2007(H5N1) |  |  |  | FJ602810 | |  | FJ602847 |  | |  |
| A/great black-headed gull/Qinghan/1/2005(H5N1) | DQ100545 | DQ100549 | DQ100553 | DQ100557 | | DQ100561 | DQ100565 | | DQ100569 | DQ100573 |
| A/gull/Germany/R882/06(H5N1) |  |  |  | AM408215 |  | | AM403148 |  | |  |
| A/herring gull/Sweden/V1116/06(H5N1) | EU889046 | EU889056 | EU889070 | EU889078 | | EU889089 | EU889100 | | EU889109 | EU122019 |
| A/slatey-backed gull/Shandong/38/04(H5N1) | DQ835807 | DQ835798 | DQ835791 | DQ188908 | | DQ835783 | DQ188910 | | DQ835777 | DQ835801 |
| A/gull/Pennsylvania/4175/83(H5N1) |  |  |  | AF082043 |  | |  | | DQ107466 |  |
| A/herring gull/NJ/402/1989(H5N3) | CY004398 | CY004397 | CY004396 | CY005926 | | CY004394 | CY004393 | | CY004392 | CY004395 |
| A/herring gull/NJ/406/1989(H5N3) | CY004976 | CY004975 | CY004974 |  | | CY004973 | CY004972 |  | |  |
| A/Sabines gull/Alaska/296/1975(H5N3) |  |  | CY015159 |  | |  | CY015157 | | CY015156 | CY015158 |
| A/gull/Delaware/4/2000(H5N4) |  |  |  |  | |  |  | | DQ107458 |  |
| A/gull/Delaware/5/2000(H5N4) |  |  |  |  | |  |  | | DQ107459 |  |
| A/herring gull/Delaware/281/98(H5N8) |  |  |  |  | |  |  | | AY664452 |  |
| A/glaucous gull/Wisconsin/486108-4/2007(H5N9) | GU051993 | GU051992 | GU051991 |  | | GU051989 |  | | GU051988 | GU051990 |
| A/ring-billed gull/DE/421733/01(H6) |  |  |  | DQ021670 | | DQ021819 |  | | DQ021750 | DQ021582 |
| A/gull/Maryland/4/77H6N1 |  |  |  |  | |  | AY207544 |  | |  |
| A/gull/Moscow/3100/2006(H6N2) | EU152234 | EU152234 | EU152236 | EU152237 | | EU152238 | EU152239 | | EU152240 | EU152241 |
| A/gull/Delaware/18/2000(H6N4) |  |  |  |  | |  |  | | DQ107415 |  |
| A/laughing gull/New York/470/2000(H6N4) |  | GU051382 | GU051381 | DQ021649 | | DQ021784 |  | | DQ021690 | DQ021590 |
| A/ring-billed gull/GA/421733/2001(H6N4) |  |  |  | GQ117282 | |  |  | |  |  |
| A/ring-billed gull/Georgia/124/2001(H6N4) | GU051389 | GU051388 | GU051387 |  | |  |  | |  |  |
| A/black-headed gull/Netherlands/1/2005(H6N8) | CY041385 | CY041384 | CY041383 | CY041378 | | CY041381 | CY041380 | | CY041379 | CY041382 |
| A/laughing gull/NJ/276/1989(H6N8) | CY004101 | CY004100 | CY004099 | CY004094 | | CY004097 | CY004096 | | CY004095 | CY004098 |
| A/gull/Italy/692-2/93(H7N2) |  |  |  | AF202248 | |  |  | |  |  |
| A/gull/Heuwiese/899-6/80H7N3 |  |  |  |  | |  | AY207515 |  | |  |
| A/laughing gull/Delaware Bay/46/2006(H7N3) | CY037094 | CY037093 | CY037092 | CY037087 | | CY037090 | CY037089 | | CY037088 | CY037091 |
| A/laughing gull/Delaware Bay/6/2006(H7N3) | CY037086 | CY037085 | CY037084 | CY037079 | | CY037082 | CY037081 | | CY037080 | CY037083 |
| A/laughing gull/Delaware/2455/2000(H7N3) | GU051494 | GU051493 | GU051492 |  | | GU051491 | GU051490 |  | |  |
| A/laughing gull/Delaware/42/06(H7N3) | EU030982 | EU030981 | EU030983 | EU030984 | | EU030985 | EU030986 | | EU030987 | EU030988 |
| A/laughing gull/NY/2455/00(H7N3) |  |  |  |  | |  |  | | DQ021689 | DQ021641 |
| A/gull/Shimane/91/88(H7N8) |  |  |  | AB270593 | |  |  | |  |  |
| A/laughing gull/DE/5/2003(H9N1) | CY004427 | CY004426 | CY004425 | CY004420 | | CY004423 | CY004422 | | CY004421 | CY004424 |
| A/Mediterranean gull/Camargue/063353/2006(H9N2) |  |  |  | EU333949 | |  | EU333950 | |  |  |
| A/laughing gull/Delaware/12/2006(H9N2) | CY041433 | CY041432 | CY041431 | CY041426 | | CY041429 | CY041428.1 | | CY041427 | CY041430 |
| A/laughing gull/DE/2718/1987(H9N5) | CY005126 | CY005125 | CY005124 | CY005986 | | CY005122 | CY014590 | | CY005121 | CY005123 |
| A/Glaucous gull/Alaska/44201-161/2006(H3N8) | HM059938 | HM059958 | HM059974 | HM060000 | | HM060005 | HM060032 | | HM060049 | HM060079 |
| A/Glaucous gull/Alaska/44198-119/2006(H6N1) | HM059928 | HM059952 | HM059981 | HM060001 | | HM060018 | HM060032 | | HM060054 | HM060078 |
| A/Glaucous gull/Alaska/44198-027/2006(H16N3) | HM059944 | HM059951 | HM059966 | HM059998 | | HM060019 | HM060028 | | HM060055 | HM060065 |
| A/Glaucous gull/Alaska/44199-097/2006(H13N3) | HM059932 | HM059959 | HM059975 | HM059995 | | HM060012 | HM060029 | | HM060046 | HM060073 |
| A/Glaucous gull/Alaska/44199-006/2006(H13N9) | HM059945 | HM059954 | HM059977 | HM059994 | | HM060016 | HM060034 | | HM060045 | HM060066 |
| A/Glaucous gull/Alaska/44199-104/2006(H13N9) | HM059931 | HM059955 | HM059967 | HM059996 | | HM060013 | HM060035 | | HM060043 | HM060072 |
