# Supplementary material for: Extensive Geographic Mosaicism in Avian Influenza Viruses from Gulls in the Northern Hemisphere
Source: PLoS One. 2011 Jun 15;6(6):e20664. doi: 10.1371/journal.pone.0020664 (PMC3115932; doi:10.1371/journal.pone.0020664)
Supplement: Table S3 — Reference sequences used to represent the American and Eurasian avian clades for the PB2, PB1, PA, NP, M, and NS segments. (DOC) [file pone.0020664.s011.doc]

**Table S3.** Reference sequences used to represent the American and Eurasian avian clades for the PB2, PB1, PA, NP, M, and NS segments.

| Virus Name | Segment GenBank Accession Number | | | | | |
| --- | --- | --- | --- | --- | --- | --- |
|  | PB2 | PB1 | PA | NP | M | NS |
| **American** |  |  |  |  |  |  |
| A/blue winged teal/TX/75/2002(H1N3) | FJ357052 | FJ357051 | FJ357050 | FJ357048 | FJ357046 | FJ375049 |
| A/blue-winged teal/ALB/293/1994 (H4N6) | CY004910 | CY004909 | CY004908 | CY004906 | CY004904 | CY004907 |
| A/mallard/Alberta/209/2003(H10N7) | CY004359 | CY004358 | CY004357 | CY004355 | CY004353 | CY004357 |
| A/mallard/California/7766/2008(H4N6) | CY039736 | CY039737 | CY039738 | CY039740 | CY039742 | CY039743 |
| A/mallard/Ohio/56/1999(H1N1) | CY012831 | CY012830 | CY012829 | CY012827 | CY012825 | CY012828 |
| A/Northern shoveler/NC/6412-052/2005(H7N6) | GU186481 | GU186480 | GU186479 | GU186477 | GU186475 | GU186478 |
| A/pintail/Alaska/310/2005(H4N6) | CY017748 | CY017747 | CY017746 | CY017744 | CY017742 | CY017745 |
| A/pintail/Ohio/454/1987(H3N8) | CY016147 | CY016146 | CY016145 | CY016143 | CY016141 | CY016144 |
| A/shoveler/ALB/114/1985(H6N2) | CY004225 | CY004224 | CY004223 | CY004221 | CY004219 | CY004222 |
| A/ruddy turnstone/DE/773/1988 (H9N6) | CY004574 | CY004573 | CY004572 | CY004570 | CY004568 | CY004571 |
| A/ruddy turnstone/Delaware/1057/2001(H10N7) | GU050954 | GU050953 | GU050952 | GU050950 | GU050948 | GU050951 |
| **Eurasian** |  |  |  |  |  |  |
| A/Duck/Hokkaido/8/80 (H3N8) | AB274963 | AB274964 | AB274965 | AB274984 | AB274986 | AB274987 |
| A/duck/Hubei/2911/2007(H5N1) | FJ784836 | FJ784820 | FJ784804 | FJ784788 | FJ784884 | FJ784868 |
| A/duck/Nanchang/1681/1992(H3N8) | CY005475 | CY005474 | CY005473 | CY005471 | CY005469 | CY005472 |
| A/mallard/ZhaLong/88/2004(H4N6) | FJ349254 | FJ349253 | FJ349252 | FJ349249 | FJ349251 | FJ349250 |
| A/ruddy shelduck/Mongolia/37/2005(H12N3) | GQ907341 | GQ907340 | GQ907339 | GQ907337 | GQ907335 | GQ907338 |
| A/duck/Novosibirsk/02/05(H5N1) | DQ864713 | DQ864714 | DQ864715 | DQ864709 | DQ864708 | DQ864707 |
| A/Eurasian wigeon/Netherlands/3/2005(H9N2) | CY043863 | CY043862 | CY043861 | CY043859 | CY043857 | CY043860 |
| A/greylag goose/Netherlands/4/1999(H6N1) | CY060195 | CY060196 | CY060197 | CY060199 | CY060201 | CY060202 |
| A/mallard/Marquenterre/Z237/1983(H1N1) | DQ864507 | DQ864502 | DQ864508 | DQ864509 | GU066781 | GU066782 |
| A/mallard/Sweden/6/2002(H2N3) | CY060363 | CY060364 | CY060365 | CY060367 | CY060368 | CY060370 |
