# Supplementary material for: Extensive Geographic Mosaicism in Avian Influenza Viruses from Gulls in the Northern Hemisphere
Source: PLoS One. 2011 Jun 15;6(6):e20664. doi: 10.1371/journal.pone.0020664 (PMC3115932; doi:10.1371/journal.pone.0020664)
Supplement: Table S4 — Availability of sequence information in the GenBank and Influenza Resource Databases for AIV from gulls. (DOC) [file pone.0020664.s012.doc]

**Table S4.** Availability of sequence information in the GenBank and Influenza Resource Databases for AIV from gulls.

| Region | Gene Segment^a^ | | | | | | | |
| --- | --- | --- | --- | --- | --- | --- | --- | --- |
|  | PB2 | PB1 | PA | HA | NP | NA | M | NS |
| American | 43 | 47 | 48 | 44 | 47 | 44 | 53 | 48 |
| Eurasian (excluding H5N1) | 16 | 15 | 14 | 52 | 23 | 18 | 20 | 19 |
| Eurasian H5N1 | 11 | 11 | 10 | 19 | 11 | 28 | 11 | 10 |
| Total | 70 | 73 | 72 | 115 | 81 | 90 | 84 | 77 |

^a^ Partial and complete sequences are included.
