# Supplementary material for: Extensive Geographic Mosaicism in Avian Influenza Viruses from Gulls in the Northern Hemisphere
Source: PLoS One. 2011 Jun 15;6(6):e20664. doi: 10.1371/journal.pone.0020664 (PMC3115932; doi:10.1371/journal.pone.0020664)
Supplement: Table S5 — Hemagglutinin and neuraminidase subtype combination frequency in viruses isolated from gull species globally. (DOC) [file pone.0020664.s013.doc]

**Table S5.** Hemagglutinin and neuraminidase subtype combination frequency in viruses isolated from gull species globally.

| NA subtype | HA subtype | | | | | | | | | | | | | | | |
| --- | --- | --- | --- | --- | --- | --- | --- | --- | --- | --- | --- | --- | --- | --- | --- | --- |
|  | 1 | 2 | 3 | 4 | 5 | 6 | 7 | 8 | 9 | 10 | 11 | 12 | 13 | 14 | 15 | 16 |
| 1 |  | 1 |  |  | **20** | 2 |  |  | 1 |  |  |  |  |  |  |  |
| 2 |  |  |  |  |  | 1 | 1 |  | 2 |  |  |  | **9** |  |  | 1 |
| 3 | 1 | 1 |  |  | 3 |  | 6 |  |  |  |  |  | 2 |  |  | **12** |
| 4 |  |  |  |  |  | 4 |  |  |  | 1 | 1 | 1 |  |  |  | 1 |
| 5 |  |  |  |  |  |  |  |  | 1 |  |  |  | 1 | 1 |  | 1 |
| 6 |  |  | 1 | 1 |  |  |  |  |  |  | 2 |  | **37** |  |  |  |
| 7 |  | 2 |  |  |  |  |  |  |  |  |  |  |  |  |  |  |
| 8 |  | 3 | 2 |  | 1 | 2 | 1 |  |  |  |  |  | 2 |  |  |  |
| 9 |  | 3 |  | 1 | 1 |  |  |  |  |  | 1 |  | **9** |  |  |  |
| ? |  |  |  |  |  | 1 |  |  |  |  |  |  | 3 |  |  |  |

The values indicate the number of viruses isolated and blank cells indicate the subtype has not been detected. The 5 most frequently identified subtype combinations are in bold.
