# Supplementary material for: Extensive Geographic Mosaicism in Avian Influenza Viruses from Gulls in the Northern Hemisphere
Source: PLoS One. 2011 Jun 15;6(6):e20664. doi: 10.1371/journal.pone.0020664 (PMC3115932; doi:10.1371/journal.pone.0020664)
Supplement: Table S6 — Identification information for AIV from the Delaware Bay gull community from 1986–1989, which form two distinct clades based on phylogenetic analyses of the PB2, PB1, PA, NP, M, and NS segments. (DOC) [file pone.0020664.s014.doc]

**Table S6.** Identification information for AIV from the Delaware Bay gull community from 1986-1989, which form two distinct clades based on phylogenetic analyses of the PB2, PB1, PA, NP, M, and NS segments.

| Name | Subtype | Year | Clade |
| --- | --- | --- | --- |
| A/laughing gull/DE/2718/1987(H9N5) | H9N5 | 1987 | 1 |
| A/herring gull/DE/698/1988(H2N1) | H2N1 | 1988 | 1 |
| A/herring gull/DE/677/1988(H2N8) | H2N8 | 1988 | 1 |
| A/herring gull/DE/692/1988(H2N8) | H2N8 | 1988 | 1 |
| A/herring gull/DE/703/1988(H2N8) | H2N8 | 1988 | 1 |
| A/herring gull/DE/670/1988(H2N9) | H2N9 | 1988 | 1 |
| A/herring gull/NJ/402/1989(H5N3) | H5N3 | 1989 | 1 |
| A/herring gull/NJ/406/1989(H5N3) | H5N3 | 1989 | 1 |
| A/laughing gull/NJ/276/1989(H6N8) | H6N8 | 1989 | 1 |
| A/herring gull/NewJersey/780/86(H1N3) | H1N3 | 1986 | 2 |
| A/herring gull/DE/475/1986(H13N2) | H13N2 | 1986 | 2 |
| A/herring gull/NJ/782/1986(H13N2) | H13N2 | 1986 | 2 |
| A/laughing gull/DE/2838/1987(H13N2) | H13N2 | 1987 | 2 |
| A/herring gull/DE/665/1988(H4N6) | H4N6 | 1988 | 2 |
| A/laughing gull/DE/554/1988(H13N3) | H13N3 | 1988 | 2 |
| A/herring gull/DE/660/1988(H13N6) | H13N6 | 1988 | 2 |
| A/herring gull/DE/712/1988(H16N3) | H16N3 | 1988 | 2 |
